# Bacterial extracellular vesicles as multifaceted regulators of human diseases and emerging therapeutic platforms

**DOI:** 10.7150/thno.132287

**Published:** 2026-05-01

**Authors:** Jing Wang, Shuwen Wang, Yanzheng Wang, Ting Zhang, Xu Zhang

**Affiliations:** 1 Department of Laboratory Medicine, School of Medicine, Jiangsu University, Zhenjiang, Jiangsu 212013, China.; 2 Affiliated Women’s Hospital of Jiangnan University, Jiangnan University, Wuxi, Jiangsu 214002, China.

**Keywords:** outer membrane vesicles, vesicle biogenesis, immune modulation, nanoplatform, biomedical applications

## Abstract

Outer membrane vesicles (OMVs) are nanoscale vesicles actively released by Gram-negative bacteria, and have become key mediators in bacterial physiology, host–pathogen interactions, and disease pathogenesis. The biogenesis of OMVs is a dynamic process, which results from membrane homeostasis imbalance, peptidoglycan remodeling, and stress responses. These mechanisms enable virulence factors, immune-regulatory molecules, and nucleic acids to be selectively or randomly loaded into vesicles. With their ability to penetrate barriers and spread throughout the body, OMVs are widely involved in the occurrence and development of a variety of human diseases. It is worth noting that the inherent characteristics of OMVs have made them a promising platform in the fields of vaccine development, cancer immunotherapy, antibacterial regulation, and disease diagnosis. This review systematically integrates the current mechanistic research and translational research results, aiming to construct a unified framework, clarify the internal relationship between the biogenesis, functional heterogeneity, and biomedical applications of OMVs, and explore the key challenges and future development directions for promoting the clinical translation of OMV-based diagnostic and therapeutic systems.

## 1. Introduction

Bacterial extracellular vesicles (bEVs) are nanoscale structures encapsulated by protein-lipid bilayer membranes, which contain a variety of biological molecules derived from the parental bacteria. Both Gram-negative bacteria and Gram-positive bacteria can actively produce bEVs and form heterogeneous vesicle subgroups with unique composition and molecular characteristics 1. Different from the early view that these vesicles are only the products of cell lysis, a large body of genetic and biochemical evidence has confirmed that they constitute a strictly regulated natural secretion pathway. It is worth noting that the biosynthesis mechanism of bEVs in Gram-negative bacteria and Gram-positive bacteria is significantly different due to the differences in their cell envelope structures.

**Figure 1:**
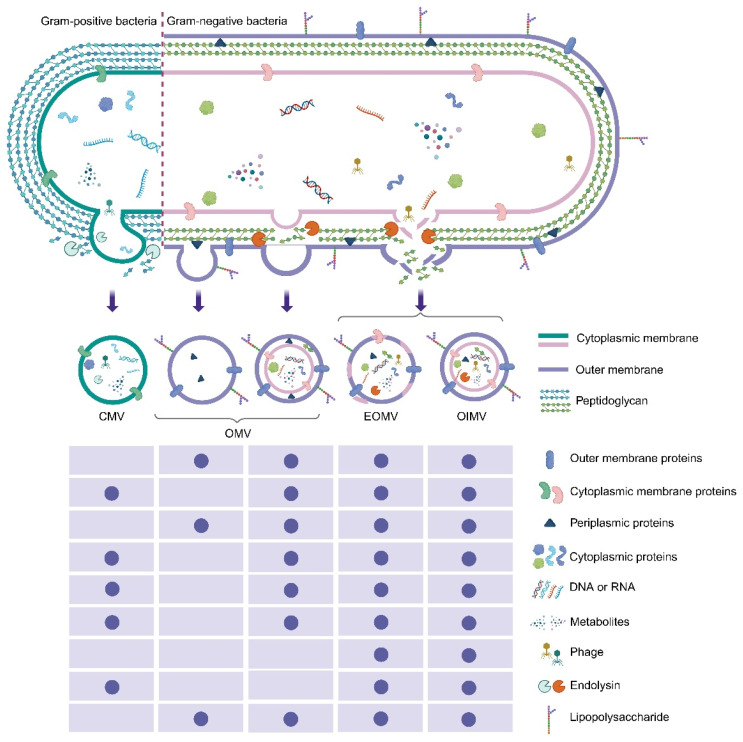
The formation of distinct membrane vesicle types. CMV: cytoplasmic membrane vesicle; EOMV: explosive outer membrane vesicle; OIMV: outer–inner membrane vesicle; OMV: outer membrane vesicle.

In Gram-negative bacteria, two main biosynthetic pathways have been identified: outer-membrane (OM) budding and explosive cell lysis. OM budding is usually driven by a disturbance of the cell envelope, such as disruptions of peptidoglycan (PG) biosynthesis or insertion of hydrophobic molecules. These changes promote the outward protrusion of the OM, and then form OMVs. During this process, the inner membrane of the bacteria remains intact, so cytoplasmic components are usually excluded from OMVs. In contrast, explosive cell lysis is triggered by phage-derived endolysin. These enzymes can degrade the PG layer and cause catastrophic rupture of the bacterial envelope. During this process, the cytoplasmic contents will be randomly wrapped, and the outer–inner membrane vesicles (OIMVs) and explosive outer membrane vesicles (EOMVs) will be formed at the same time **(Figure 1)** 2.

In Gram-positive bacteria, the formation of cytoplasmic membrane vesicles (CMVs) is usually attributed to the activity of endolysin or other PG-degrading enzymes. The destruction of local PG structure causes the cytoplasmic membrane to bulge outward and form vesicles, and finally produces the CMVs that encapsulate membrane components and cytoplasmic cargo (**Figure 1**). In addition, the formation of tube-shaped membranous structures (TSMSs) is believed to be due to the focal degradation of PG, which expands the membrane structure and subsequently forms nanotubes. These TSMSs may promote intercellular communication and coordination in the process of biofilm formation and dispersion. It is worth noting that bEVs can also be derived from phage endolysin-mediated cell lysis. In Gram-positive bacteria, these vesicles have been shown to mediate the transfer of phage receptors from sensitive bacteria to drug-resistant bacteria, thus temporarily expanding the host range of phages. In addition, the acclimated defective prophage residues can actively regulate the production of bEVs to enhance host adaptability, especially under environmental pressures such as low pH or high osmolarity. In general, these vesicle structures play a multidimensional role in bacterial communication, host-microbial interaction, and host immune regulation, and affect the pathogenicity and symbiotic outcomes.

This review systematically summarizes the research progress of OMVs, a typical bacterial extracellular vesicle, focusing on its emerging insights in the fields of biosynthetic mechanism, disease pathogenesis, and biomedical applications, and discusses the current challenges and future development opportunities in this field. Although OMVs have inherent analytical and mechanism complexity, related research has greatly deepened our understanding of bacterial physiology and provided a theoretical basis for the development of innovative treatment strategies, thus accelerating the process of clinical transformation.

## 2. Biogenesis of OMVs

As a typical representative of bEVs, OMVs are spherical nanostructures with diameters ranging from 20 to 250 nm. The membrane structure is composed of bilayer lipids derived from the OM of Gram-negative bacteria: the outer leaflet is lipopolysaccharide (LPS), and the inner leaflet is phospholipid. OMV biogenesis is a multifaced regulatory process. Extensive research over the past decades has progressively clarified the cargo-sorting principles and the conditions under which cytoplasmic components gain access to OMVs. Existing evidence supports two general kinds of vesicle formation: (i) vesiculation through the outward budding and scission of intact bacterial OMs, and (ii) vesicle release associated with membrane rupture and explosive cell lysis 2.

**Figure 2:**
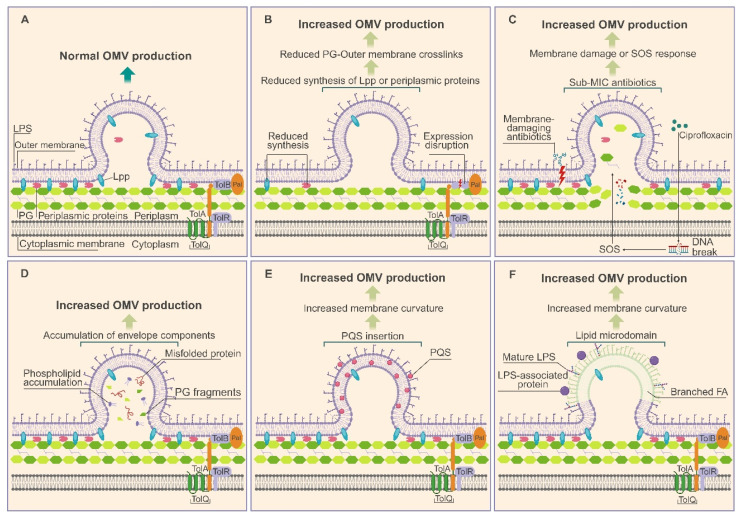
Current models of OMV biogenesis. (A) Normal OMV biogenesis. (B) Attenuation of OM and PG cross-linking proteins, including reduced synthesis of Lpp and periplasmic proteins or disruption of the Tol-Pal complex, drives OM curvature and promotes OMV formation. The Tol-Pal complex (TolA, TolB, TolQ, TolR, Pal) spans the envelope and interacts non-covalently with PG to maintain membrane stability. (C) Antibiotic-induced envelope stress enhances OMV production. Exposure to Sub-MIC antibiotics can cause DNA damage and activate the SOS response, which alters LPS synthesis and modifies OM composition, thereby promoting OMV formation. (D) In regions where misfolded proteins or envelope components such as LPS or PG fragments accumulate, cross-linking structures are either displaced or locally depleted, promoting bulging of these OM nanodomains and resulting in increased OMV production. (E) Insertion of PQS molecules into OM leaflets can increase membrane curvature, thereby promoting OMV formation. (F) Lipid microdomains enriched in specific LPS, phospholipids, or LPS-associated molecules exhibit outward bulging due to charge, cargo, or membrane fluidity, promoting OMV production. FA: fatty acid; Lpp: lipoprotein; LPS: lipopolysaccharide; OM: outer membrane; OMVs: outer membrane vesicles; PG: peptidoglycan; PQS: *Pseudomonas* quinolone signal; Sub-MIC: sub-minimum inhibitory concentration. Peptidoglycan degradation plays a central role in OMV formation via explosive cell lysis. Phage-derived endolysins cleave the PG layer, causing catastrophic loss of cellular integrity and the release of vesicles containing randomly incorporated cytoplasmic content. In *Shewanella vesiculosa* M7T (*S. vesiculosa* M7T), prophage-activated lysis generates a heterogeneous population of membrane-bound vesicles, including explosive OIMVs 8. Antibiotic stress can cause similar effects. Ciprofloxacin-treated *Stenotrophomonas maltophilia* (*S. maltophilia*) produces conventional OMVs. It also produces larger OIMVs that contain many cytoplasmic proteins and have a filament-like shape.

Disruption of the structural linkages between the OM and the PG layer is well known to be a driver of vesicle formation. Breakage or attenuation of these cross-linking proteins gradually detach the OM, promoting its curvature and eventual vesiculation. Yu et al. demonstrated that CRISPR-dCas9–mediated downregulation of *pbpC*, a key determinant of PG integrity, and *wbpP*, a gene involved in LPS biosynthesis, markedly reduces OM–PG crosslinking and yield the highest OMV production reported to date 3. Similarly, deletion of *rmpM* in *Neisseria meningitidis* (*N. meningitidis*), encoding a protein that anchors the OM to the PG layer, significantly enhances OMV release **(Figure 2)** 4.

Perturbations to membrane homeostasis alone can also stimulate vesiculation. Disruption of *tolB*, a gene essential for maintaining membrane integrity in *Helicobacter pylori* (*H. pylori*), results in a substantial increase in OMV secretion. In *Acinetobacter baumannii* (*A. baumannii*), mutation of the *ctp* gene destroys the stability of the cell envelope and interfere with PG biosynthesis, resulting in excessive production of OMVs 5. Antibiotics that destroy membrane integrity, such as polymyxin B and colistin, also trigger significant formation of OMVs. It is worth noting that the latest research shows that the excessive formation of OMVs is not limited to membrane-damaging agents. Antibiotics with sub-inhibitory concentrations targeting different cell pathways, including meropenem and ceftazidime (PG synthesis inhibitors), chloramphenicol and tigecycline (translation inhibitors), and ciprofloxacin (DNA replication inhibitor), can significantly stimulate the secretion of OMVs of *Escherichia coli* 47EC (*E. coli* 47EC), indicating that the biogenesis of OMVs is a conserved stress adaptation strategy** (Figure 2)** 6.

The imbalance of membrane component biosynthesis also drives the formation of OMVs. In *E. coli*, the simultaneous knockout of the *nlpI* and *mlaE* genes that regulate cell envelope structure and phospholipid distribution synergistically induces the excessive formation of OMVs due to the accumulation of phospholipids in the outer leaflet 7. OMV biogenesis can additionally be triggered by the insertion of curvature-inducing molecules into the OM. The best-characterized example is found in *Pseudomonas aeruginosa* (*P. aeruginosa*), where the quorum-sensing molecule *Pseudomonas* quinolone signal (PQS) intercalates into the outer leaflet to induce membrane curvature and drive OMV formation while promoting its own selective packaging **(Figure 2)**.

## 3. OMVs in bacterial physiology

Although the production of OMVs appears to be an energy-consuming process, they are conserved in different bacterial species, which indicates that the physiological advantages conferred by the biosynthesis of OMVs exceed their metabolic costs 8. Therefore, it is of great significance to explore the functions of OMVs to reveal the adaptive mechanisms of bacteria.

OMVs promote the survival, stress tolerance and toxicity of bacteria by coordinating various physiological processes. One of the core functions is to relieve cell envelope stress. They can selectively remove misfolded proteins and abnormal membrane components to maintain the stability and integrity of the outer membrane. Bacteria can also affect the structure and function of OMVs by adjusting the lipid composition, so as to accurately control the fluidity and mechanical strength of the membrane. For example, *Pseudomonas syringae* (*P. syringae*) regulates vesicle properties by enriching fatty acids that promote membrane curvature 9; *P. aeruginosa* adds rigid phospholipids to enhance the membrane structure 10; *Salmonella enterica* (*S. enterica*) can dynamically adjust the content of LPS in OMVs according to environmental signals to adapt to external changes and reduce the risk of being recognized by the host immune system 11, 12. In general, the release and composition regulation of OMVs constitute the key mechanisms for bacteria to maintain membrane homeostasis, manage stress and regulate virulence.

In addition to their role in remodeling membrane structure, OMVs also play an important role in host–pathogen interactions and bacterial communication. They act as multifunctional delivery vectors, mediating complex interactions with the host by delivering virulence factors and immune regulatory molecules. For instance, enterotoxigenic *E. coli* transports heat-labile enterotoxin within OMVs to the host Golgi apparatus, triggering electrolyte efflux and concurrently activating both pro-inflammatory and immunosuppressive pathways. OMVs also serve as "long-range weaponry" of bacteria to establish ecological colonization sites by promoting tissue invasion, destroying host cell function, and subverting immune defense 13. In the multi-microbial community, they coordinate the dynamics of biofilms in the form of structural scaffolds rich in DNA and adhesins, and play a role by delivering matrix-degrading enzymes that promote dispersion. These biofilm structures further enhance the resistance of bacteria to environmental stresses such as antibiotics 14. It is worth noting that the development stage of bacteria affects the regulation mechanism mediated by OMVs: for example, the death-phase extracellular vesicles released by *P. aeruginosa* disintegrate the mature biofilm through iron-dependent ferroptosis, while the growth-phase vesicles promote the expansion of biofilm, which highlights the dual regulatory role of EVs in biofilm homeostasis 15.

Bacteria have also evolved sophisticated strategies that rely on OMVs to achieve immune escape and nutrition acquisition. For example, *Salmonella* upregulates the expression of outer membrane protein PagC by activating the PhoPQ regulatory pathway, so as to promote the production of OMVs. These PagC-enriched OMVs have special functions in the host: they can act as "complement bait", actively bind to complement component C3b, and recruit the host complement inhibitor factor H. Then, factor H promotes the conversion of C3b into inactive iC3b, blocking opsonization and the formation of the membrane attack complex. Through this ingenious mechanism, *Salmonella* can transfer the complement-mediated innate immune attack from its own surface and effectively neutralize it, thus avoiding serum killing and cell lysis 16. At the same time, bacteria selectively load iron carriers into OMVs to obtain scarce iron resources 17. This process is mediated by specific TonB-dependent iron carrier receptors, which are significantly enriched in the OMVs. The process of protein transport into vesicles also depends on special transport systems, especially the Sec complex, which is responsible for selectively introducing functional proteins into the internal cavity of vesicles or localizing them to the vesicle membrane. This selective loading mechanism is not fixed, but controlled by a complex regulatory network regulated by environmental signals. Specifically, when bacteria are in the environment of iron deficiency in plant extracellular space, genes related to iron carrier transport (e.g., Cluster II) are significantly activated and up-regulated. Through this regulation, bacteria can secrete OMVs rich in iron carriers, which can spread over a wide spatial range and actively chelate free iron ions in the surrounding environment. Subsequently, the receptors on the surface of bacteria can recognize and retrieve these iron carrier complexes, so as to realize the efficient recycling of iron resources. Taking *P. syringae* as an example, this iron acquisition strategy based on OMVs enables it to break through the host plant's nutritional restriction defense (especially the iron deficiency defense mechanism), and maintain its growth in an iron-deficient environment, thus occupying a significant advantage in competition with other microorganisms 17.

In summary, although the formation of OMVs requires a lot of energy, their diverse functions jointly improve the adaptability of bacteria by supporting bacterial survival in complex environments, promoting interspecific competition, and enhancing virulence. Therefore, the formation of OMVs is a precisely regulated process, and their precise control of cargo selection and release confers significant evolutionary advantages on bacteria.

## 4. Multifaceted roles of OMVs in human diseases

OMVs are nanoscale carriers released by Gram-negative bacteria, which are rich in pathogen-associated molecular patterns (PAMPs), virulence factors, nucleic acids, and other effector molecules. As a powerful medium for interkingdom communication, OMVs are involved in the pathogenesis of a variety of diseases. Accumulating evidence suggests that they may be involved in gastrointestinal disorders, neurodegenerative diseases, respiratory diseases, cardiovascular diseases, tumor development, systemic diseases such as diabetes mellitus, and bone metabolism abnormalities. Their pathogenesis has multiple convergence characteristics, including toxic cargo delivery, pattern-recognition receptor signal activation, physiological barrier destruction, and immune response regulation. It is worth noting that because OMVs can not only cause local inflammation and tissue damage, but also circulate through the bloodstream, they may act as potential remote amplifiers contributing to the onset and progression of diseases and the formation of complications. The specific pathogenic role of OMVs in different diseases will be described in detail below.

### 4.1 OMVs in gastrointestinal diseases

OMVs play an important role in the pathogenesis of gastrointestinal diseases through three interrelated axes: inflammatory activation, disruption of epithelial barrier integrity, and the promotion of tumorigenesis. Pathogenic *E. coli*–derived OMVs, enriched in enterohemolysin, induce mitochondrial-targeted apoptosis in intestinal epithelial cells while activating caspase-11–dependent noncanonical inflammasomes, leading to DNA double-strand breaks and diarrheal disease 18, 19. Similarly, OMVs from enterohemorrhagic *E. coli* O157 induce strong IL-8 production through TLR4/5-NF-κB signaling, which contributes to diarrhea-associated hemolytic uremic syndrome 20. Beyond classical enteric infections, bacterial components of the gut microbiome, especially OMVs, have emerged as potential signaling molecules involved in shaping the inflammatory microenvironment of primary sclerosing cholangitis-associated inflammatory bowel disease 21. In dysbiotic states, OMVs derived from the gut microbiota of high-protein-fed mice further exacerbate colitis by activating epithelial TLR4 signaling, which amplifies secretory IgA responses and promotes an inflammatory environment 22.

However, the role of OMVs in inflammation is dichotomous, as commensal-derived vesicles often orchestrate protective responses. *Bacteroides fragilis* (*B. fragilis*)-derived OMVs relieve inflammation by delivering miR-5119 to target host PD-L1, which downregulates Gasdermin D and thereby prevents the formation of neutrophil extracellular traps and facilitates the growth of intestinal stem cells 23. OMVs from the human gut commensal *Alistipes timonensis* (*A. timonensis*) also delay colitis progression by actively transporting immunomodulatory sulfonolipids from the gut into the systemic circulation 24. At the same time, *Bacteroides*-derived sphingolipid-enriched OMVs, which mainly contain dihydroceramide phosphoethanolamine, activate the host mevalonate pathway and induce the secretion of IL-10 by dendritic cells (DCs), which may contribute to strengthening the anti-inflammatory immune response 25. Host factors further play a regulatory role in this mutually beneficial symbiotic relationship. In the presence of microbiota, mammalian intestinal epithelial cells secrete apolipoprotein L9a/b (APOL9a/b) and its human homolog APOL2. These proteins selectively target *Bacteroides* symbionts by directly binding to microbial ceramide-1-phosphate. APOL9 deposition does not directly exert bactericidal activity, but triggers a membrane stress response, resulting in excessive release of OMVs by targeted symbionts. These OMVs derived from *Bacteroides* are then recognized by DCs through the TLR2-MyD88 signaling pathway, which promotes multicellular interaction with intraepithelial lymphocytes (IELs) to produce IFN-γ. This signal axis induces intestinal epithelial cells to express major histocompatibility complex class II (MHC-II) molecules, and ultimately promotes the development of CD4^+^CD8αα^+^ IELs, potentially enhancing the immune barrier function of the intestinal tract against intestinal pathogens 26.

In addition to regulating the immune response, the OMVs secreted by intestinal pathogens may also contribute to their environmental adaptation and immune escape in the host. Take* S. enterica* as an example. The OMVs released by *S. enterica* are rich in PagC protein. These OMVs can recruit host Factor H and hydrolyze C3b to inactive iC3b, potentially acting as "complement bait" and "guiding" complement from the surface of the bacteria 16. At the same time, these OMVs can bind to and sequester cationic antimicrobial peptides such as polymyxin B through electrostatic and hydrophobic interactions, thereby forming a protective barrier. Under the condition of excessive vesicle production induced by β-lactam antibiotics, this protective effect may be further amplified, so that this kind of tolerance can be "shared" or transferred to adjacent bacteria at the functional level 27. The situation of *Vibrio cholerae* (*V. cholerae*) is similar. After entering the host, the bacterium will rapidly reshape its surface structure through the release of OMVs. This process is triggered by the silencing of the transcription of the VacJ/Yrb (Mla) ABC transport system, resulting in a large number of OMVs 28. On the one hand, excessive vesiculation helps to "physically shake off" some unfavorable components (such as porin OmpT) from the cell surface, so as to adapt to the intestinal environment containing bile salts faster; on the other hand, these vesicles may serve as protective carriers of active cholera toxin, which can not only prevent the toxin from being degraded by intestinal protease, but also continuously and effectively deliver cholera toxin into host cells through caveolin-mediated endocytosis 28, 29.

In terms of barrier integrity, OMVs derived from *Fusobacterium nucleatum* (Fn-OMVs) disrupt epithelial homeostasis by activating the TLR4–ERK/CREB/NF-κB signaling pathway and trigger the secretion of IL-8 and TNF-α 30. The OMVs of *V. cholerae* can destroy the intestinal epithelial barrier by delivering HapA protease, a zinc-dependent metalloproteinase. HapA can specifically target and degrade key tight-junction-related proteins such as ZO-1, claudin and β-catenin, thereby weakening the integrity of intercellular junction structure 31. In contrast, beneficial OMVs may help support the resilience of epithelial cells. For example, OMVs from *Faecalibacterium prausnitzii* (*F. prausnitzii*) reprogram gut microbiota metabolism to elevate phosphatidylcholine, thus suppressing the RIPK1-RIPK3-MLKL signaling pathway and potentially reducing epithelial cell necroptosis when the animal is infected with porcine epidemic diarrhea virus 32.

In addition to inflammation and barrier dysfunction, OMVs may also influence gastrointestinal tumorigenesis. Fn-OMVs are enriched in colorectal cancer tissues and transfer adhesin FomA to the host membrane, facilitating interaction with bacterial FN1441 protein to enhance autoaggregation and tumor colonization 33. In the stomach, *H. pylori* OMVs transport CagA and VacA toxins to the gastric mucosa and cause excessive IL-1β and TNF-α production, thereby promoting the progression from chronic gastritis to gastric cancer 34. Studies have found that the OMVs secreted by some symbiotic bacteria may exert anti-tumor protective effects under environmental stress, and this protective effect is closely related to diet structure and mental health—these factors will significantly affect the composition and function of intestinal microbiota. For example, *Akkermansia muciniphila* (*A. muciniphila*) may inhibit the growth of colorectal tumors induced by chronic stress by secreting protective OMVs. However, continuous psychological stress and other environmental pressures will damage the integrity of intestinal mucosa and make the mucous layer thinner. The mucus layer is not only the main habitat of *Akkermansia* bacteria, but also an important source of nutrition. Therefore, this destruction may lead to a significant decline in the number of bacteria and the abundance of their associated vesicles. In their respective growth processes, *A. muciniphila* actively and selectively loads up to 226 specific proteins, enzymes and lipids into the vesicles, making these OMVs complex and precise bacteria-host communication carriers. When environmental stress inhibits the secretion of OMVs, the protective interaction between flora and host may be weakened. This not only reduces the ability of vesicles to inhibit the proliferation of colorectal cancer cells after internalization, but also weakens its function of regulating tumor immune microenvironment through TLR2/NLRP3 and other signaling pathways. These studies suggest a potential link between the gut-brain-microbiota axis and tumor inhibition: environmental stressors can change the anti-tumor protective effect mediated by OMVs by remodeling the intestinal microenvironment 35.

### 4.2 OMVs in neurodegenerative diseases

OMVs are increasingly recognized as potential systemic mediators connecting peripheral infection and pathological changes of the central nervous system. In Alzheimer's disease (AD), gingipains (Rgp and Kgp) concentrated in *Porphyromonas gingivalis*-derived OMVs (Pg-OMVs) may promote the degradation of tight junction proteins (especially ZO-1 and occludin) in cells after internalization (such as entering cells through clathrin-dependent endocytosis). The hydrolysis of these proteins may increase the permeability of the blood-brain barrier (BBB) 36. At the same time, Pg-OMVs associated LPS can trigger microglial neuroinflammation by activating TLR4-MyD88 signaling axis, and then recruit p-AKT and p-JNK pathways to participate in signal transduction, thereby potentially upregulating the expression of inducible nitric oxide synthase (iNOS) and TNF-α 37.

In addition to the inflammatory response initially triggered, OMVs are assumed to be able to reprogram the neuroimmune microenvironment more finely. For example, chronic exposure to these vesicles is thought to induce microglia to form a hypersensitive phenotype, which is characterized by *de novo* expression of MHC-II molecules and upregulation of complement receptors (CR1, CR3, CR4). This phenotypic change may make microglia act as a "danger sensor". It is speculated that this change will not only amplify the release of cytokines (TNF-α, IL-1β, IL-6), but also recruit more activated microglia to gather in the injured area through C3a/C5a signaling 38. In the hippocampus, these cytokines induced by OMVs have been suggested to act as upstream regulators that may activate the GSK-3β pathway. This pathway is closely related to the hyperphosphorylation of tau protein and the consequent synaptic loss 39.

Consistent with the "double-edged sword" hypothesis, OMVs, acting as concentrates of virulence factors, participate in the pathogenesis process on the one hand, and may also trigger the host's protective defense response to a certain extent. More and more new evidence shows that amyloid-beta (Aβ), which has long been regarded as solely neurotoxic, may initially play a role in the innate immune system in the form of an antimicrobial peptide, which is used to capture and neutralize pathogens delivered by OMVs 38. Under this theoretical framework, neurodegenerative changes are regarded as a maladaptive host defense outcome: under the continuous pressure of chronic adventitial vesicle stimulation, these protective proteins may undergo pathological accumulation, potentially promoting the disease progression.

The systemic role of OMVs is thought to extend to other pathogens through a variety of mechanisms. In the mouse model, *Aggregatibacter actinomycetemcomitans* OMVs (Aa-OMVs) have been reported to deliver extracellular RNA and activate the TLR8/NF-κB axis, thereby potentially promoting the production of TNF-α 40. Similarly, OMVs derived from gut dysbiosis have been suggested to aggravate AD-like pathological changes by activating NF-κB signaling pathway and stimulating IL-6 secretion in the hippocampus. In contrast, specific gut commensal-derived OMVs have demonstrated neuroprotective potential via the gut-brain axis. For instance, OMVs derived from *A. muciniphila* can alleviate smoking-induced cognitive impairment and synaptic loss. These OMVs serve as essential vehicles to deliver the tryptophan metabolite indole-3-lactic acid to the brain, where it activates aryl hydrocarbon receptor signaling in microglia. This interaction effectively reprograms microglial metabolism from a pro-inflammatory glycolytic state toward oxidative phosphorylation, thereby suppressing neuroinflammation and restoring cellular bioenergetics 41. In Parkinson's disease models, it is reported that *H. pylori*-derived OMVs (Hp-OMVs) can bypass the tight junctions of gastric epithelial cells and cross the BBB. It is speculated that Hp-OMVs may promote the migration and proliferation of microglia by activating p38 MAPK signaling pathway, which may contribute to the loss of dopaminergic neurons 42.

Neuron loss is also increasingly considered to be associated with ferroptosis mediated by OMVs. Ferroptosis is a regulatory cell death process driven by iron-dependent lipid peroxidation. OMVs may initiate this cascade reaction by inhibiting the expression of the cystine-glutamate exchanger (System Xc-) and downregulating the expression of its subunits SLC7A11 and SLC3A2. This inhibition may lead to the depletion of glutathione in cells, and then inactivate glutathione peroxidase 4 (GPX4), which is the core regulator responsible for clearing lipid peroxides, and further lead to the accumulation of lipid reactive oxygen species (ROS) to lethal levels in cells 43. At the same time, the upregulation of key ferroptosis-promoting factors related to OMVs, such as acyl-CoA synthase long chain family member 4 (ACSL4), prostaglandin-endoperoxidase synthase 2 (PTGS2) and NOX1, may further amplify this oxidative damage process. At the ultrastructural level, neurons undergoing OMV-induced ferroptosis may exhibit typical pathological changes of mitochondria, such as mitochondrial membrane rupture, loss of cristae structure and increased membrane density 44. In addition, the neural immune reprogramming induced by OMVs, especially the infiltration of CD8^+^ T cells and the release of IFN-γ, may further improve the sensitivity of neurons to death signals. This OMV-activated IFN-γ signaling has been suggested to inhibit the System Xc-pathway at the transcriptional level through the STAT1/IRF-1 axis, thus further supporting a mechanistic link between OMVs and neurodegenerative cell death at the molecular level 43. In general, these findings highlight the role of OMVs as potential contributors within the microbe-gut-brain axis, suggesting that they may be involved in the onset and progression of neurodegeneration through multiple pathways, such as synergistically destroying the blood-brain barrier, reprogramming the neuroimmune system, and influencing pathological protein aggregation.

### 4.3 OMVs in respiratory diseases

OMVs play a variety of pathogenic roles in respiratory diseases by directly inducing inflammation and tissue damage, and indirectly regulating host defense mechanisms and microbial community dynamics. In acute respiratory infections, *Klebsiella pneumoniae*-derived OMVs (Kp-OMVs) potently activate NF-κB signaling in bronchial epithelial cells, promoting the phosphorylation and nuclear translocation of the p65 subunit, and the p65 subunit binds to the IL-8 promoter, leading to a robust upregulation of IL-8 expression, thus inducing inflammatory responses associated with respiratory infection 45. At the same time, Kp-OMVs can also induce mitochondria-dependent apoptosis in epithelial cells, which is characterized by the upregulation of pro-apoptotic proteins BAX and BIM, the inhibition of the anti-apoptotic factor Bcl-xL, and the activation of the caspase-9/-3 cascade, accompanied by endoplasmic reticulum stress and oxidative damage, which together may aggravate lung tissue injury 46. In the process of acute infection, OMVs mainly play the role of a "first strike", by triggering pro-inflammatory reactions and a surge in cytotoxicity, which can contribute to the disruption of the host's physical barrier, thereby creating favorable conditions for the rapid colonization and spread of pathogens to surrounding tissues.

In addition to directly causing tissue damage, pathogens also use OMVs as protective shields to evade the attack of the innate immune system. For example, in the acidic airway environment of patients with bronchiectasis, *P. aeruginosa* OMVs induce the overproduction of 2-heptyl-4-quinolone, which can interfere with the membrane binding affinity of antimicrobial peptides such as LL-37, thereby enhancing the continuous colonization ability and drug resistance of bacteria 47. During *Legionella pneumophila* (*L. pneumophila*) infection, both bacterial and host-derived extracellular vesicles may contribute to immune regulation in a coordinated manner. Bacterial OMVs trigger Toll-like receptor 2 (TLR2) signaling on uninfected macrophages to induce pro-inflammatory cytokine production and neutrophil/macrophage recruitment for pathogen removal; meanwhile, host-derived exosomes selectively target alveolar epithelial cells to release IL-6, CXCL8, GM-CSF, and MCP-1. Through differentiated cell targeting, OMVs and exosomes may synergistically regulate the inflammatory cascade and immune clearance dynamics during the course of pneumonia.

*Pneumophila*'s OMVs show a time-dependent immunoregulatory ability in the occurrence of Legionnaires' disease. Their effects on the host immune system at different stages are not fixed: in the early stage of infection, OMVs can activate and promote the host defense response; with the development of the disease, their role may gradually change to help bacteria escape the surveillance of the immune system, so as to realize immune escape. In the early stage of infection (within 24 hours), these OMVs, as powerful pro-inflammatory stimulants, are recognized by macrophages mainly through TLR2 receptors, quickly trigger the nuclear translocation of the p65 subunit, induce the activation of classical macrophages and secrete a large number of cytokines such as IL-8, IL-6 and TNF-α. This surge of immune response may temporarily inhibit the replication of intracellular bacteria. The same signaling pathway also upregulates miR-146a (a key negative regulator of innate immune response) through transcription, potentially laying the groundwork for subsequent immune escape. With the progress of infection, the increase of miR-146a level leads to the continuous degradation and translation inhibition of kinase IRAK-1. This depletion of IRAK-1 mediated by OMVs desensitizes macrophages, significantly reduces their response to subsequent bacterial stimulation, and shifts the immune balance from the activated state to the inhibited state. At the same time, OMVs may promote the survival of host cells by upregulating anti-apoptotic signals (such as BCL2A1 targets), and avoid premature cell death when the bacterial load is too high. At 48 hours after infection, this change from a restrictive microenvironment to a permissive microenvironment can significantly increase the number of vacuoles of *L. pneumophila* in each cell, thus creating more optimized conditions for the long-term survival and dissemination of bacteria 48. Importantly, such endogenously derived, vesicle-mediated communication is also therapeutically exploitable. Oral administration of PEGylated probiotics generates IL-1Ra-enriched OMVs that are able to cross the gut-vascular barrier and accumulate selectively in the lungs, where they may suppress the macrophage pyroptotic process and attenuate the dysregulated inflammation in septic lung injury 49. Furthermore, Pg-OMVs derived from the gut commensal have demonstrated significant potential in alleviating acute lung injury via the gut-lung axis. These vesicles increase systemic cholic acid levels by modulating bile acid metabolism and reshaping the gut microbiota composition, which effectively suppresses lung macrophage pyroptosis through the inhibition of the NF-κB signaling pathway 50.

Unlike the acute phase characterized by rapid and severe tissue destruction, in chronic respiratory diseases, OMVs may adopt a more subtle and refined "regulatory" strategy, which relies on long-term and gradual weakening of the host's innate clearance function and reshaping the interactions between different microorganisms to maintain the stable ecological niche required for the long-term survival and colonization of pathogens in the respiratory tract. In pulmonary fibrosis, specific Gram-negative bacteria such as *Bacteroides* and *Prevotella* that are overgrown promote fibrotic progression through OMV-induced pro-fibrotic effects. OMVs induce IL-17B production by alveolar macrophages and recruit neutrophils and Th17 cells, which sustains chronic pulmonary inflammation and fibrotic remodeling 51. Environmental exposure may exacerbate OMV-caused damage: Gram-negative bacterial EVs in household dust are easily internalized by airway epithelial cells and alveolar macrophages, activate innate immune reactions through LPS-TLR4 signaling, lead to Th1/Th17 polarization, and result in neutrophilic airway inflammation.

OMVs additionally facilitate chronic respiratory infection by impairing host defense mechanisms and reshaping microbial interactions. In pulmonary cystic fibrosis, *Pseudomonas aeruginosa* (*P. aeruginosa*) OMVs (Pa-OMVs) carrying cystic fibrosis transmembrane conductance regulator (CFTR) inhibitors may hijack the host's ubiquitination pathway by stabilizing the inhibitory interaction between G3BP1 and the deubiquitinating enzyme USP10. In this way, the recycling process of CFTR is blocked, and its protein is more easily degraded by lysosomes, potentially weakening the mucociliary clearance system, a key defense mechanism responsible for long-term pathogen clearance, rather than just causing transient cell death. Similarly, in the airways of patients with bronchiectasis, Pa-OMVs induce excessive production of 2-heptyl-4-quinolone, interfering with the binding of antimicrobial peptides to bacterial membranes, thereby enhancing bacterial resistance and promoting the chronic persistence of pathogens. Unlike the characteristics of competitive expansion and rapid destruction in the acute infection stage, in the context of chronic disease progression, OMVs tend to shape an ecological environment that is conducive to the long-term survival of pathogens 52. Similarly, *Moraxella catarrhalis* (*M. catarrhalis*) relies on the special complement escape mechanism mediated by OMVs to improve its colonization ability in the respiratory tract. These OMVs carry bacterial surface proteins UspA1 and UspA2, which can directly bind complement component C3 in a non-covalent manner, thus blocking the activation of complement cascade. OMV-mediated C3 "isolation" can form a protective microenvironment locally, which not only helps *M. catarrhalis* escape the clearance of the immune system, but also provides a barrier against immune attack for other pathogens co-colonized, especially *Haemophilus influenzae* 53. In chronic obstructive pulmonary disease, the relationship between *M. catarrhalis*-derived OMVs (Mc-OMVs) and host IL-1β pathway is not a simple activation, but a synergistic amplification. The activation effect of Mc-OMVs alone on human β-defensin 2 (hBD-2) was relatively weak. However, on the premise of the existing IL-1β signal, the effect of Mc-OMVs is significantly amplified, which will promote epithelial cells and neutrophils to secrete hBD-2 in large quantities and cooperatively. At the same time, OMVs can also induce apoptosis of lung epithelial cells, which may be mediated by the interaction between vesicle-related uspa1 and CEACAM1 on the surface of host cells, thereby weakening the bronchial mucosal barrier function and promoting the invasion of bacteria to deeper tissues 54. The strategic shift of OMVs from acute destruction to chronic regulation of host deubiquitination and dynamic changes in complement system fully demonstrates their diversity and adaptability in different disease backgrounds.

### 4.4 OMVs in atherosclerosis

OMVs can potentially break through the anatomical barrier that prevents maternal bacteria from entering the human body, which is considered to be a key early driver of atherosclerosis. It is worth noting that even in a healthy state, these nanoscale vesicles may still have the ability to penetrate. They can move along the paracellular pathways, or be engulfed by DCs and pass through the complete oral or intestinal epithelium, and finally enter the systemic circulation. Once in the blood, OMVs become concentrated carriers of LPS and virulence factors (such as gingival protease secreted by *P. gingivalis* and CagA of *H. pylori*) 55. These factors are more destructive than free LPS in inducing endothelial dysfunction. Such early "molecular attack" may activate the ROS/NF-κB signaling pathway, cause the re-expression of adhesion molecules (ICAM-1, VCAM-1 and E-selectin) and chemokines (CXCL1, CXCL2, CXCL8), so as to recruit monocytes to the vascular endothelium. In addition, OMVs can directly damage the physical defense of the endothelium by destroying the glycocalyx, a skeletal structure crucial to vascular homeostasis. The latest evidence shows that Pg-OMVs promote vascular endothelial glycocalyx injury through the PPAD/CitH3/B3GAT1 pathway. During this process, OMVs-related peptidylarginine deaminase translocates into the nucleus and induces histone H3 citrullination, thereby inhibiting the expression of glycosyltransferase B3GAT1 and reducing key glycocalyx components. This structural degradation not only increases vascular permeability, but also significantly enhances monocyte adhesion to the endothelium, thus accelerating the progress of atherosclerosis 56. Correspondingly, the gingipains carried in the OMVs released by *P. gingivalis* may specifically cleave the endothelial junction protein CD31, which can not only destroy the structural integrity of the vascular barrier, but also potentially increase the penetration of lipid and inflammatory cells into the vascular intima, thereby contributing to the progress of atherosclerosis 55.

With the progress of the disease, the role of OMVs has also changed from a local pathogenic factor to a systemic amplifier. Through the "remote regulation" mechanism mediated by OMVs, the development of existing pathological processes could be accelerated. For example, after the transport of CagA-carrying OMVs and host-derived extracellular vesicles to distant atherosclerotic plaques, the lipid homeostasis of macrophages can be destroyed by downregulating the transcription factors PPARγ and LXRα. This interference could inhibit the expression of cholesterol efflux transporters (ABCA1, ABCG1), lead to abnormal accumulation of cholesterol and promote the formation of foam cells 57. In addition, OMVs from *E. coli* and *P. aeruginosa* could promote the transfer of LPS to the host cytoplasm, trigger the death of macrophages by activating the caspase-11 pathway, and further expand the core of lipid necrosis 58. The OMVs released by P. gingivalis could also induce Runx2-dependent calcification in vascular smooth muscle cells by activating ERK signaling pathway, which might make arterial plaques more unstable 55.

At the stage of plaque rupture, OMVs may contribute to thrombosis through direct and indirect mechanisms. Directly, OMVs could act as a powerful procoagulant stimulator, interact with platelet surface receptors, and directly induce platelet activation and aggregation. For example, Pg-OMVs can induce significant platelet aggregation and degranulation without relying on its parent bacteria, and promote the release of α and dense granules. Similarly, OMVs from *Meningitidis* might facilitate the formation of platelet-leukocyte complexes, thereby accelerating the formation of local microthrombosis 55. Indirectly, OMVs may exacerbate the thrombogenic environment by shifting the endothelial surface toward a pro-coagulant state. For example, enterotoxigenic* E. coli* OMVs have been shown to induce endothelial cells to upregulate the expression of tissue factor and downregulate the level of thrombomodulin, so as to create a more conducive microenvironment for coagulation 55. In addition, the previous destruction of endothelial cell connections by OMVs could expose subendothelial connective tissue, providing the necessary physical support for the rapid adhesion and activation of platelets after plaque rupture. Therefore, OMVs are not only biomarkers in the process of infection, but also may play a role as contributors to the inflammatory reaction of atherosclerosis and subsequent thrombosis.

### 4.5 OMVs in cancer

As the key medium of intercellular communication between bacteria and host cells, OMVs play a complex role that may contribute to cancer through a variety of molecular mechanisms during tumor initiation, progression, and metastasis. A typical feature of their tumor-promoting activity is the activation of oncogenic signaling pathways. For example, Fn-OMVs may activate the Wnt/β-catenin pathway through FadA protein binding to the EC5 domain of E-cadherin, resulting in the upregulation of Wnt target genes and pro-oncogenic inflammatory mediators, thus promoting the development of oral squamous cell carcinoma (OSCC) and colorectal cancer (CRC) 59. At the same time, Fn-OMVs can also activate the TLR4/NF-κB axis and induce the production of IL-8, TNF, and other inflammatory cytokines 30. Similarly, Hp-OMVs may trigger NF-κB signal transduction, enhance IL-8 secretion, and stimulate macrophages to release oncostatin M, which together could contribute to gastric cancer progression 30. Hp-OMVs also deliver virulence factors, including CagA and VacA, directly into host cells, promoting gastric carcinogenesis through genetic perturbations and precancerous lesion formation. Beyond classical toxin-mediated effects, the aryl hydrocarbon receptor and FOS signaling axis has been suggested as a potential mediator of OMV-induced cellular injury. *A. baumannii* OMVs may activate host aryl hydrocarbon receptor (AHR) by inducing tryptophan-2,3-dioxygenase (TDO) to produce the ligand kynurenine, which could subsequently drive FOS-mediated cytotoxicity and promotes a pro-tumorigenic environment 60.

In addition to activating oncogenic signaling, OMVs may influence tumor cell differentiation and phenotypic plasticity. Co-culture of CRC cells with OMVs derived from *Campylobacter jejuni* (*C. jejuni*) or non-pathogenic commensal *E. coli* upregulates genes associated with cellular differentiation, a phenomenon similarly observed with *Vibrio cholerae* (*V. cholerae*) OMVs. Notably, while most OMV–host interactions promote tumorigenesis, certain OMVs exhibit context-dependent anti-tumor activities. For example, the sublytic activity of the pore-forming protein cytolysin A (ClyA), packaged within OMVs from non-pathogenic *E. coli*, has been shown to suppress colon cancer cell growth by modulating the EZH2/miR-622/CXCR4 signaling axis, epigenetically downregulating oncogenic proteins and restoring expression of the tumor suppressor p53 61.

More prominently, OMVs have been reported to induce epithelial–mesenchymal transition (EMT) and enhance invasive phenotypes. Fn-OMVs trigger EMT in OSCC cells, promoting migration, invasion, and lung metastasis in vivo 59. Fn-OMVs also facilitate bladder cancer lymphatic metastasis through their most abundant outer membrane protein, FomA. Mechanistically, FomA-containing OMVs directly engage TLR2 to trigger the NF-κB signaling pathway and upregulate IL-6, which induces M2b macrophage polarization and the subsequent release of vascular endothelial growth factor C 62. Pg-OMVs further enhance tumor aggressiveness through various mechanisms, including inhibiting ferroptosis and promoting EMT via NF-κB in OSCC 63, and OMV-enriched small RNAs (sRNAs) such as sRNA23392 may directly enhance OSCC invasion and migration by targeting the adhesion molecule desmocollin-2 64.

In the tumor microenvironment (TME), OMVs act as microbe-related molecular patterns and may initiate tumor-promoting inflammatory responses through interactions with Toll-like receptors and other pattern recognition receptors. For example, Pg-OMVs or LPS engage Siglec-7 on human monocyte-derived DCs, which leads to inflammatory and tumor-supportive phenotypes 65. Further supporting OMV-mediated immune suppression, Fn-OMVs have been observed to potentially confer resistance to cancer immunotherapies by delivering exogenous tryptophanase to tumor-associated macrophages (TAMs) to activate the TDO2/AHR axis and subsequently induce the expression of immunosuppressive cytokines and immune checkpoints, which could limit the infiltration of cytotoxic T lymphocytes (CTLs) into the TME 66. While native OMVs often foster such immunosuppressive niches, engineered OMV platforms are being developed to counteract these effects, particularly in the context of therapeutic resistance. For instance, engineered OMVs (STM-Mn@OMVs) have been shown to reverse m6A methylation-based immunosuppression occurring after insufficient radiofrequency ablation of hepatocellular carcinoma. By inducing tumor cell pyroptosis and synergistically activating the STING pathway, these modified OMVs effectively promote DC maturation and T-cell activation, offering a promising strategy to remodel the suppressive TME 67.

OMVs also appear to upregulate vascular endothelial growth factor expression and may activate its receptors within the TME, promoting angiogenesis and tumor proliferation 68. Importantly, circulating OMVs disseminate systemically via lymphatic and hematogenous routes, suggesting the possibility of remodeling of distant organs. By reprogramming myeloid cells and propagating pro-inflammatory signals, OMVs may contribute to the formation of pre-metastatic niches, and could help promote metastatic spread 69.

Although some OMVs exhibit antitumor activities (such as inducing apoptosis, inhibiting proliferation, or activating antitumor immunity), the current evidence largely supports their pro-tumorigenic roles. Nevertheless, the functional landscape of OMVs in cancer remains incompletely defined. Systematic investigation is required to delineate the specific effector molecules, downstream signaling pathways and context-dependent roles of different kinds of OMVs in various tumors, as well as to explore their potential diagnostic and therapeutic potential.

### 4.6 OMVs in metabolic musculoskeletal and reproductive disorders

As a key mediator of bacteria–host interaction, OMVs play multidimensional roles in a variety of systemic diseases. Taking metabolic diseases such as diabetes as an example, Pg-OMVs enter the circulatory system and disrupt the integrity of vascular endothelium by promoting the formation of stress fibers and inducing the endocytosis and degradation of VE-cadherin, thereby increasing vascular permeability, and potentially aggravating diabetic vascular dysfunction associated with *Porphyromonas gingivalis* infection 70. At the same time, Pg-OMVs also impair hepatic glucose metabolism through a gingipain-dependent suppression of insulin-stimulated Akt/GSK-3β signaling, which may contribute to hyperglycemia and disease progression 71. This capacity for systemic dissemination extends to barrier-spanning inflammatory axes. Notably, OMVs derived from the gut commensal *Parabacteroides goldsteinii* traverse the intestinal barrier to modulate the gut–skin axis, effectively suppressing epidermal hyperplasia and systemic inflammation in psoriasis 72. Beyond these systemic axes, engineered OMVs have shown significant potential in addressing the microinflammation that drives chronic kidney disease. By utilizing gut-derived *B. fragilis* OMVs modified with kidney-targeting peptides to deliver anti-IL-1β single-chain antibodies, it is possible to precisely target damaged proximal renal tubules. This localized delivery effectively reduces the expression of pro-inflammatory cytokines such as TNF-α and MCP-1, thereby mitigating tubular inflammation and alleviating associated renal injury 73.

OMVs exert dichotomous and context-dependent effects on bone remodeling and musculoskeletal homeostasis. OMVs from periodontal pathogens (e.g., *Filifactor alocis*, *P. gingivalis*, *Tannerella forsythia*) and commensal *Streptococcus oralis* activate TLR2 via lipoprotein/LPS engagement, triggering pro-inflammatory cytokine release, osteoclastogenesis, and bone resorption, thereby driving pathological bone loss 74, 75. More specifically, Pg-OMVs hinder bone regeneration by inducing ferroptosis in bone marrow mesenchymal stem cells via activation of the Hippo-YAP signaling pathway 76. However, some OMVs have protective effects against bone loss. *Proteus mirabilis* OMVs (Pm-OMVs) downregulate miR-96-5p to upregulate the expression of Abca1 and activate the mitochondrial apoptosis pathway, thereby potentially inhibiting the differentiation of osteoclasts. Similarly, *Kingella kingae* OMVs (Kk-OMVs) also inhibit macrophage-to-osteoclast differentiation, which may attenuate osteoarticular lesions. To complement the biological observations above, a macrophage-targeted nanoparticle strategy, which rejuvenates aged pro-inflammatory macrophages and restores macrophage phagocytosis, represents a promising approach to restrain inflammation-mediated bone resorption in aging-related skeletal disorders 77.

In inflammatory joint diseases, OMVs contribute directly to synovial inflammation and tissue destruction. In rheumatoid arthritis (RA), Fn-OMVs deliver the adhesin FadA into joint cavities, activating Rab5a GTPase and the inflammatory regulator YB-1 in synovial macrophages, thereby amplifying local inflammatory cascades and exacerbating joint destruction 78. Pg-OMVs further exacerbate RA by promoting immune evasion and increasing inflammation of the synovium 79. In addition, Pg-OMVs also modulate *Staphylococcus aureus* (*S. aureus*) infection in RA by inducing reversible bacterial aggregation, facilitating neutrophil internalization, and rendering bacteria resistant to neutrophil-mediated killing.

OMVs also exert potentially significant regulatory effects on reproductive health and pregnancy outcomes. The reprogramming of peritoneal macrophages using OMV-coated poly (lactic-co-glycolic acid) (PLGA) nanoparticles has been shown to shift the peritoneal microenvironment toward an anti-fibrosis M1 phenotype, thereby inhibiting myofibroblast activation and preventing the progression of endometriosis 80. In the field of male infertility, *E. coli* OMVs, which are often detected in the semen of infertile men, may damage sperm function by reducing progressive motility, increasing the proportion of immobile sperm, elevating intracellular ROS levels, and potentially aggravating DNA fragmentation 81. High concentrations of Pm-OMVs can induce sperm membrane remodeling, mitochondrial hyperpolarization, and accumulation of ROS, thereby activating autophagy and apoptosis pathways and potentially further weakening sperm motility 82.

OMVs also play potentially important roles in pregnancy-related and developmental disorders. Pg-OMVs disrupt trophoblast-endothelial interaction and modulate trophoblast-neutrophil immune communication, and may impair placental vascular transformation and immune homeostasis. *H. pylori* OMVs are able to cross the placental barrier and interfere with thymic T cell development in the offspring 83. After internalization by placental trophoblasts, Pg-OMVs have been reported to significantly inhibit glycolytic activity, reduce the production of reactive oxygen species (ROS), maintain mitochondrial function, and induce a metabolically quiet state in trophoblasts to restrict placental and fetal growth 84. Animal experiments demonstrate that maternal exposure to Pg-OMVs during gestation results in significantly reduced brain weight in offspring, accompanied by microglial activation and decreased cortical neuronal density, indicating that Pg-OMVs can potentially alter embryonic brain development and suggesting that *P. gingivalis* may influence postnatal development through multiple mechanisms 85.

**Figure 3:**
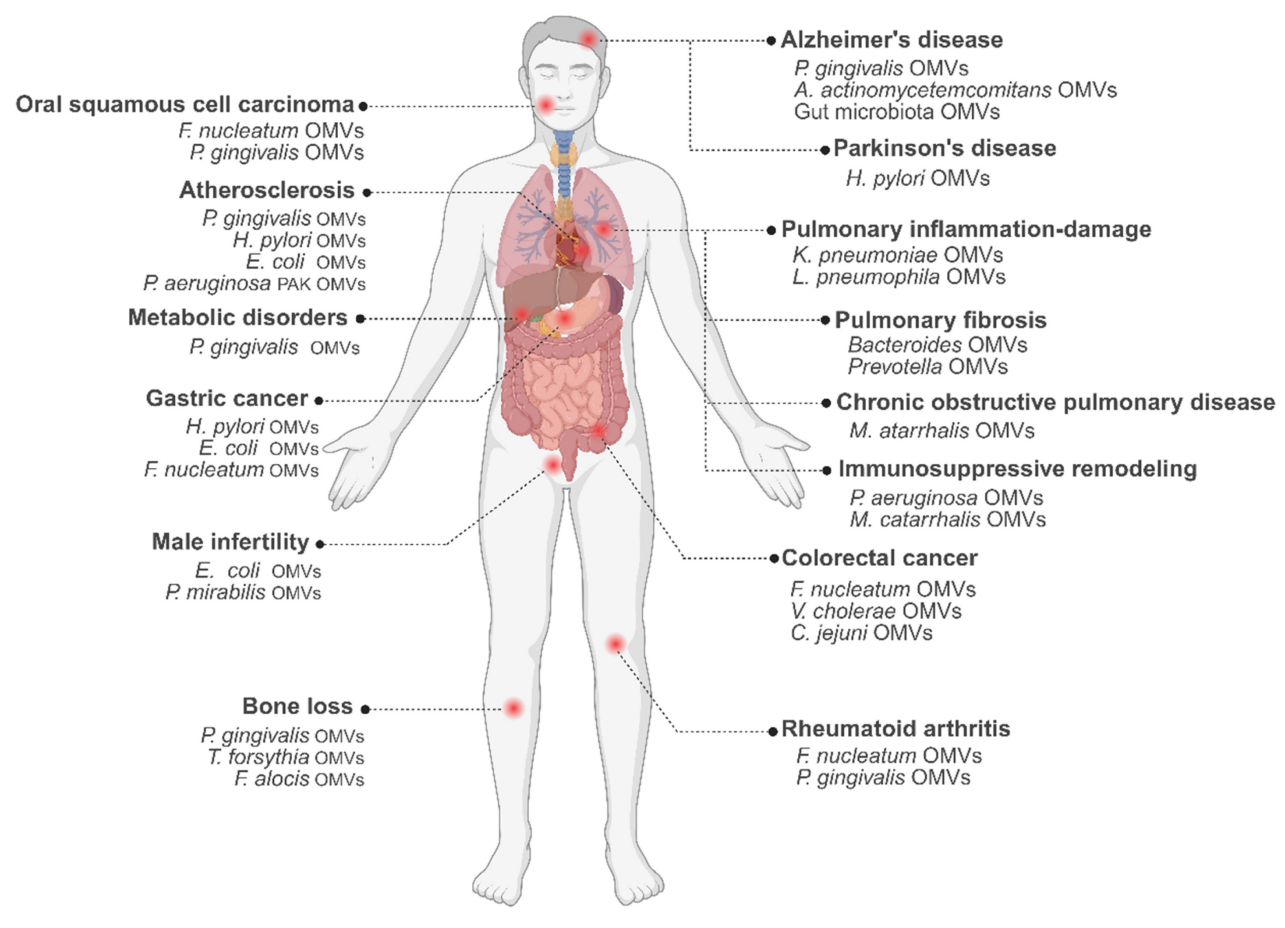
The pathological roles of OMVs in human diseases.

Collectively, these findings underscore the diverse and system-wide pathogenic mechanisms by which OMVs influence metabolic, musculoskeletal, reproductive, and developmental disorders **(Figure 3)**.

## 5. Potential applications of OMVs in disease diagnosis and therapy

### 5.1 OMVs as safe and potent vaccines

#### 5.1.1 The potential of native and engineered OMVs as vaccines

OMVs possess considerable inherent advantages as a vaccine platform 86. As naturally occurring, non-replicating spherical nanoparticles derived from Gram-negative bacteria, OMVs show high immunogenicity, mainly due to their composition that contains antigenic determinants as well as abundant PAMPs. Because of the presence of antigen-presenting capability as well as intrinsic adjuvant characteristics, OMVs have a very strong immunostimulatory capacity. In their particulate structure formed by phospholipids, LPS, outer membrane proteins, and enclosed periplasmic contents, strong innate and adaptive immune reactions are induced 87. Moreover, the moderate nanosize of OMVs (typically ranging from 20 to 250 nm in diameter) promotes their efficient uptake by antigen-presenting cells (APCs) and enables their free drainage into lymph nodes, thereby regulating the intensity and polarization of immune activation 87. Unlike conventional vaccines that require complex inactivation or attenuation procedures, OMVs can be easily biologically engineered to express heterologous antigens with their native conformational epitopes, a significant characteristic to generate high-titer specific antibodies. Research indicates that presenting protein antigens within a native-like OMV environment may endow them with distinct structural dynamics compared to soluble forms. For instance, evidence suggests that OMV-embedded meningococcal Neisseria adhesin A antigen appears to be more susceptible to trimer opening and may display a larger antigenic surface, a configuration linked to the induction of antibodies with superior bactericidal activity 88. Importantly, the OMV platform can not only effectively stimulate humoral immunity but also induce a Th1-type dominant cellular immune response. OMV have a stable structure and can maintain its integrity under high temperature and chemical conditions. These characteristics bring significant advantages for vaccine storage and transportation 89. In a word, by combining antigen delivery with the characteristics of self-adjuvanticity, OMVs represent a multi-functional vaccine platform with promising safety and immunogenicity, showing potential as a new generation of vaccine carriers.

Although promising therapeutically, the clinical translation of natural OMVs is hampered by inherent immunotoxicity and low production yields. In order to overcome these deficiencies, a variety of engineering approaches have been proposed to improve their safety and therapeutic effects. Genetic engineering represents a central approach for attenuating immunotoxicity. For instance, flagellin deletion and removal of the main phosphate group of LPS produce engineered OMVs (EMVs) with much lower inflammatory activity. The EMVs reduce circulating TNF-α and IL-6 in mice more than 10-fold and 15-fold, respectively, while maintaining excellent biocompatibility 90. Similarly, recombinant OMVs from the endotoxin-free *ClearColi* strain induce balanced Th1/Th2 humoral responses as well as DC maturation and provide complete protection against lethal influenza challenge in animal models. Chemical engineering approaches, such as NA-conjugated peptidoglycan inhibitors, can overcome yield bottlenecks and increase OMV production by up to 65-fold via a single coculture step. By inhibiting peptidoglycan formation to induce membrane protrusion, this universal platform also enables custom functionalization of OMVs with promising applications in photodynamic cancer treatment 91.

**Figure 4:**
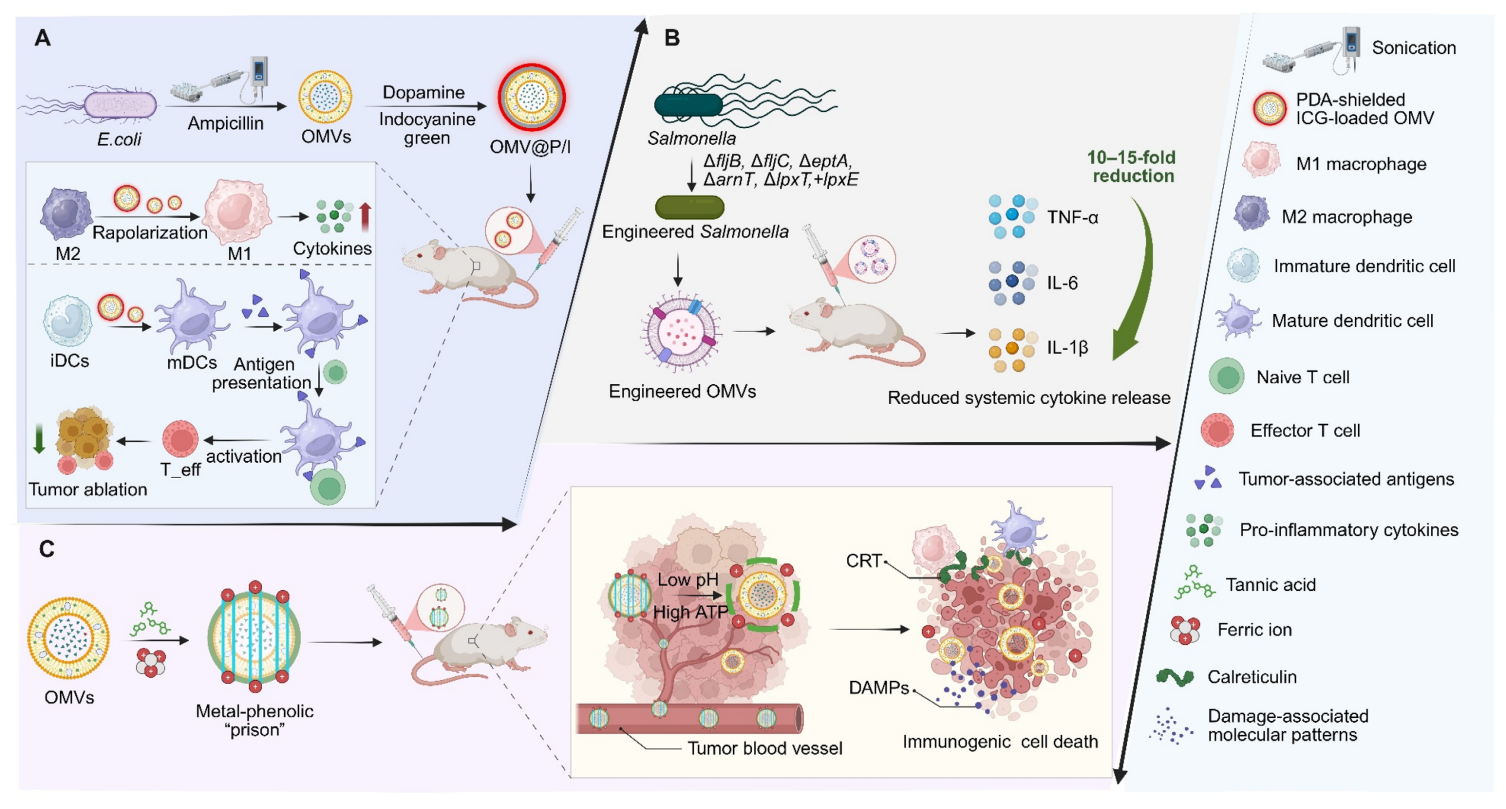
Engineered OMVs enable safe immune activation and tumor microenvironment remodeling. (A) PDA-coated OMVs loaded with indocyanine green induce near-infrared irradiation-triggered tumor killing and enhance antitumor immunity by promoting M2-to-M1 macrophage repolarization, dendritic cell maturation, and T-cell activation. (B) Engineered OMVs exhibit greatly reduced inflammatory activity. (C) Systemically administered metal-polyphenol-detoxified OMVs are released in the acidic, ATP-rich TME upon shell dissociation. The liberated OMVs remodel the TME by repolarizing TAMs from M2 to M1, and act as potent adjuvants that combine with tumor antigens and DAMPs from ICD to form an in situ vaccine, thereby inducing robust antitumor immunity. CRT: calreticulin; DAMPs: damage-associated molecular patterns; iDCs: immature dendritic cells; ICD: immunogenic cell death; mDCs: mature dendritic cells; OMVs: outer membrane vesicles; PDA: polydopamine; TAMs: tumor-associated macrophages; TME: tumor microenvironment.

Surface engineering provides an additional strategy to mitigate LPS-associated toxicity. Coating OMVs with a biocompatible polydopamine (PDA) shell masks LPS, and enables TME reprogramming without observable adverse effects 92. Alternatively, constructing a metal–phenolic network “cage” on the OMV surface allows pH- and ATP-responsive disassembly within the TME, thereby minimizing systemic toxicity while locally releasing iron ions to trigger immunogenic cell death (ICD) and enhance tumor antigen presentation **(Figure 4)** 93.

In addition, alternative bacterial sources provide another promising strategy. OMVs from non-pathogenic bacteria, such as *Caulobacter crescentus* (*C. crescentus*), represent a potential new-generation vaccine platform because of their naturally weak inflammatory response characteristics and negligible in vivo toxicity 94.

#### 5.1.2 Optimizing OMV production for scalable manufacturing

Engineering strategies for OMVs are also aimed at improving yield to enable large-scale production. Genetic engineering is a key approach, such as the construction of the proteome-minimized *E. coli* strain BL21(DE3)Δ60, in which 59 endogenous proteins have been deleted, significantly increasing OMV production and yields more than 40 mg of vesicles per liter of culture 95. Physical and chemical induction methods also significantly increase the production of OMVs. Using ampicillin pretreatment combined with ultrasonic disruption technology can increase the vesicle yield by up to 40-fold compared with traditional extraction methods 92. Also, stimulating OMV release using exogenous phage lysins—such as LysP53—may further boost vesicle protein yield and improve OMVs purity 96.

### 5.2 Engineered OMVs as multifunctional platforms for cancer immunotherapy

#### 5.2.1 OMVs as natural adjuvants for antitumor immune activation

OMVs have emerged as a highly promising platform for cancer immunotherapy owing to their inherent immunostimulatory capacity. As natural adjuvants that strongly activate both the innate and adaptive immune systems, OMVs have been found to help DCs mature and become activated and also make APCs process and present a wide variety of antigens, causing a strong adaptive immune response 97, 98. This cascade, in turn, activates and facilitates the intratumoral infiltration of both antigen-specific and tumor-specific T cells and further initiates the cGAS-STING signaling pathway. Additionally, outer membrane proteins in OMVs recruit cytotoxic T lymphocytes (CTLs) and natural killer cells into tumor tissues and stimulate the massive secretion of interferon-γ (IFN-γ), potentially increasing antitumor immunity.

#### 5.2.2 OMV mediated TME reprogramming

OMVs actively remodel the immunosuppressive tumor microenvironment through complementary spatial, metabolic, and immunomodulatory mechanisms. Nanoreactors such as MnO₂@OxA@OMV mitigate tumor hypoxia and oxidative stress by catalyzing H₂O₂ to O₂ 99, while biomimetic fusion membranes (hyaluronidase-decorated OMVs fused with PD-L1–knockout cancer cell membranes) exploit neutrophil hitchhiking to bypass dense extracellular matrices, facilitating deep tumor penetration and enhancing antigen delivery 100. Furthermore, a legumain-responsive nanoinhibitor platform has been engineered by surface-conjugating a PD-L1 blockade peptide onto OMVs and applying a protective PEG coating. This modification ensures safe intravenous delivery and enables enzyme-triggered release in the TME to recruit innate immune cells, effectively transforming "cold" tumors into "hot" ones while preventing T-cell exhaustion 101. OMVs can also convert immune “cold” tumors into “hot” tumors by regulating macrophage metabolism 102, inducing apoptosis- and autophagy-mediated immunogenic cell death 103, and promoting macrophage repolarization from the M2 to the M1 phenotype 104. CpG-loaded mesoporous silica nanocomposites coated with OMVs (CpG@MSN-PEG/PEI@OMVs) can stimulate DC maturation, restore T-cell metabolic fitness, and sustain long-lasting tumor-specific immune memory 105.

Targeted metabolic and signaling interventions have been developed, including OMVs regulating the IRG1–itaconic acid axis to enhance CXCL9/10 secretion and CD8⁺ T-cell infiltration while delivering PD-L1 nanobodies 106, and biomineralized OMVs (OMVs@MnCaP-FA) activating the cGAS–STING pathway and modulating lactate metabolism 107. To specifically address therapeutic resistance, engineered OMVs delivering lactate oxidase have been utilized to target and deplete lactate within the TME. By neutralizing acidic stress, these OMVs effectively radiosensitize tumor cells and improve immune cell infiltration, offering a synergistic strategy to overcome radiotherapy tolerance 108. STING-agonist nanobody OMVs targeting cadherin 17 combined with photoimmunotherapy and CD47 blockade eradicate metastatic lesions and reinforce immune memory 109. Chimeric nanozyme OMVs (OMV-DFA) catalyze glucose depletion and ferroptosis while releasing tumor-associated antigens to synergize with intrinsic OMV immunomodulation 110.

Recent innovations further enhance macrophage-based cytotherapy and in situ OMV production. C16-ceramide-fused OMVs (RILO) harboring TLR7/8 agonists and IDO1 inhibitors target GPC3-targeted macrophages, reprogram TAMs and boost T-cell cytotoxicity in glypican-3-positive tumors 111. Engineered bacteria act as in vivo “pharmaceutical factories” that continuously produce GM-CSF and SIRPα siRNA, while OMVs protect the siRNA and enable a sustained transition of TAMs toward an M1-like phenotype, thereby sustaining tumor suppression 112. To counteract the nutrient deficiency, OMV-coated L-arginine nanomicelles loaded in adoptive macrophages serve as metabolic depots, sustaining the pro-inflammatory phenotype and increasing cytotoxicity toward solid tumors 113. All these OMV-based platforms, together with spatial targeting, metabolic reprogramming, and immune modulation, offer potential strategies for breaking the immunosuppressive environment and enhancing antitumor immunity.

#### 5.2.3 OMV based combination therapeutic platforms for synergistic tumor eradication

OMVs have emerged as highly adaptable platforms for combination cancer therapies, which can be integrated with physical, chemical, genetic, and immunological modes to achieve potentially synergistic tumor eradication. Physical modality–based combinations have been extensively explored. For instance, HPDA@[OMV-CC] nanoparticles combine photothermal therapy (PTT) with the intrinsic immunogenicity of OMVs to achieve precise targeting of melanoma, promote the maturation of DCs, and eliminate the tumor with no detectable systemic toxicity 114. Likewise, biomimetic cascade-targeting nanosystems such as siRNA@PLOV are produced by fusing photosensitive thermosensitive liposomes (PTSLs) with attenuated *Salmonella* OMVs. Sequential targeting of tumors and intratumoral T-cells occurs, reprogramming the tumor immune microenvironment 115. Extending these strategies to radiotherapy, hafnium–phenolic–coated OMVs (Hf-OMVs) function as high-Z radiosensitizers while alleviating hypoxia through catalase-mediated oxygen generation, collectively enhancing tumor antigen release, DC activation, and systemic antitumor immunity against both primary and distant lesions 116.

Aside from physical interventions, the OMV-based combination platform has been increasingly engineered to induce immunogenic tumor cell death and stimulate immune responses. The “CPApoptosis” nano-actuator (OCT@ES), which encapsulates the copper transporter elesclomol within Cu–tannic acid–modified OMVs, triggers a synergistic cascade of cuproptosis, non-canonical pyroptosis, and oxidative apoptosis, effectively converting immunologically “cold” tumors into “immune-on” states and markedly enhancing PD-L1 blockade efficacy 117. In parallel, synthetic bacterial vesicles loaded with STING agonists robustly activate DCs and interferon signaling, increase tumor-infiltrating T cells, and elicit durable antitumor responses in melanoma and colon cancer models with a favorable safety profile 118.

Complementing these approaches, OMVs have been integrated with biological agents and immune cell–mediated delivery strategies. Neutrophil-hitchhiking OMVs carrying anti-CD47 antibodies and chlorin e6 (NOC47-Ce6), take advantage of the post-operative inflammation for site-specific release, and upon illumination, it improves the tumor antigen display, switches TAMs to a pro-inflammatory state, restores phagocytosis, and suppresses the residual tumor growth as well as metastasis 119. OMVs have also been used to potentiate oncolytic virotherapy, with the oncolytic virus OH2 encapsulated in *E. coli* Nissle 1917 (EcN)–derived OMVs, improving viral stability and intratumoral accumulation while reprogramming immunosuppressive TAMs via OMV-associated PAMP signaling 120.

Since genetic engineering and modular modification can be achieved more quickly, self-assembled OMVs containing dCas9–ClyA–sgRNA complexes can be used for efficient and multiplexed gene delivery. OMVs co-delivering CXCL9 and IL-12 (OMV-C9I12) transform tumor cells into cytokine-secreting niches to attract effector T-cells via JAK–STAT and work with immune checkpoint blockade to overcome resistance in multiple solid tumor models. 121. Expanding OMV-based combination strategies onto immune-privileged sites, OMV-C-C, that co-display cell-penetrating peptides with chlorotoxin, is able to get into and selectively target glioblastoma, after which CD8⁺ T-cells are drawn in and induce IFN-γ-dependent ferroptosis via the inhibition of cystine-glutamate exchange and downregulation of glutathione peroxidase 4 43.

Finally, hybrid vesicle and delivery technologies further broaden the therapeutic versatility of OMV-based systems. Eukaryotic–prokaryotic hybrid vesicles produced by the fusion of membranes and carrying indocyanine green (ICG) show potent synergistic antitumor effects in conjunction with local PTT 122. Bacteria–plant hybrid nanovesicles, made from thylakoid and OMVs, show strong tumor tropism, amplify immune responses, and inhibit tumorigenesis and metastasis. In parallel, non-invasive delivery strategies like iontophoresis-driven dual microneedle patches (IPMN-G and IPMN-C) can facilitate the entry of antigens and chemokines into the skin via transdermal transport, potentially boosting DC activation as well as lymph node homing, and enhancing the performance of the transdermal immunization platform 123.

#### 5.2.4 Engineered OMVs for next-generation tumor vaccines and precision delivery

To enhance antitumor efficacy, extensive efforts have leveraged genetic engineering and surface modification to transform OMVs into advanced tumor vaccine carriers and immunotherapeutic platforms. A representative strategy is the construction of single-dose prophylactic or personalized tumor vaccines through the combination of bacterial OMVs and tumor cell membranes to achieve efficient antigen presentation and strong immune stimulation 97, 98. OMVs also show a good synergistic effect with immune checkpoint inhibitors. This can be achieved by engineering OMVs to deliver PD-1–encoding plasmids, enabling tumor cell–intrinsic PD-L1 blockade 124, or by displaying PD-1 directly on the surface for tumor-targeting combined with checkpoint inhibition 125. A biosynthetic, ultrasound-triggered *in situ* vaccine (OMVs^GM^-Lip@Ce6) has been engineered to further enhance checkpoint therapy by integrating GM-CSF–expressing bacterial OMVs with dual-responsive liposomes. In the acidic tumor microenvironment, this hybrid platform appears to facilitate localized GM-CSF release to recruit dendritic cells, while subsequent ultrasound-activated immunogenic cell death promotes the release of tumor-associated antigens. This coordinated mechanism, leveraging intrinsic OMV adjuvanticity alongside cytokine delivery, has demonstrated potential in remodeling the immunosuppressive TME and overcoming acquired resistance to PD-1 blockade in refractory models 126. Extending immunization to in situ vaccination, glycine-induced OMVs (Gomv) have been developed as an inhaled vaccine for metastatic lung cancer. They have improved production yield, decreased endotoxin content, and enriched immunogenic outer membrane proteins. After pulmonary administration, Gomv targets alveolar macrophages and triggers FPR1/2-NF-κB-dependent M1 polarization and tumor phagocytosis, thereby activating cytotoxic T cells and achieving robust tumor suppression 127.

In addition to vaccination, OMVs can also serve as versatile vectors for gene therapy, including gene silencing by small interfering RNA (siRNA). They can also be used in combination regimens with chemotherapeutic agents, such as doxorubicin or paclitaxel, and other nucleic acid-based therapeutics, such as microRNA. This flexibility is also highlighted by the development of advanced biomimetic nanoplatforms 102, 125. These include hybrid constructs integrating OMVs with photosynthetic bacteria for tumor antigen redirection 128, as well as dual-functional vesicles formed by fusing immunogenic probiotic OMVs with cellular membranes overexpressing anti–PD-L1 single-chain variable fragments. When labeled with near-infrared II probes, these systems enable high-resolution TME imaging and amplify photothermal–immunotherapy by promoting M2-to-M1 macrophage repolarization and enhancing local CD8⁺ T-cell infiltration 129.

In parallel, immune cell–mediated delivery strategies have been developed, such as neutrophil-affinitive OMVs camouflaging PLGA nanoparticles carrying photosensitizers and TGF-β inhibitors, which exploit neutrophil hitchhiking to tumors, induce N1 polarization, and trigger elastase-mediated tumor cell killing while reversing immunosuppression 130. Complementary approaches employ hyaluronidase-expressing OMVs modified with 3-aminophenylboronic acid to enable CD73 siRNA delivery, degrade hyaluronic acid–rich stroma, suppress CAF activity, and inhibit pro-angiogenic signaling within the TME 131.

Further expanding therapeutic versatility, OMVs have been integrated into multifunctional hybrid systems and autonomous delivery paradigms. Macrophage microrobots composed of OMVs, magnetic nanoparticles, and macrophages have been developed for multimodal therapy 132, self-propelled micromotors to carry out both mechanical disruption and immunostimulation 133, and ZIF-8–based hybrids for combined miRNA delivery and PD-1 presentation 125. In order to overcome the immune resistance and blood-brain barrier of glioblastoma, Angiopep-2-modified OMV platforms have been developed to co-deliver doxorubicin and CD47 siRNA, thereby enabling sustained penetration across the BBB and reprogramming macrophages and microglia to enhance therapeutic efficacy 134. More importantly, a transformative strategy uses engineered bacteria as an “in vivo cell factory” to continuously assemble RNA-loaded OMVs in a self-assembled manner, which could load mRNAs, miRNAs, or siRNAs directly into the host. A single dose of bacteria producing PD-L1 siRNA-packed OMVs induced substantial gene silencing and tumor regression, emphasizing the possibility of translation of self-reproducing OMV biomanufacturing 135.

Collectively, these advances position OMVs as versatile and programmable platforms for tumor vaccination and immunotherapy, enabling integrated antigen presentation, immune modulation, and therapeutic delivery through genetic engineering, biomimetic design, and autonomous in vivo production. Such modular and scalable OMV-based systems point to a promising direction for next-generation cancer immunotherapy **(Table 1)**.

### 5.3 Multifaceted functions of OMVs in antimicrobial and virulence modulation

#### 5.3.1 The dynamic ecological role of OMVs in microbiota crosstalk and disease evolution

The function of OMVs is not only to support the survival of a single strain, but also to act more like a universal "biological currency", which can regulate the complex interactions between strains at different stages of disease evolution. Prior to the onset of clinical infection, OMVs released by symbiotic bacteria play a key role in maintaining the stability of microbial populations and achieving competitive exclusion. For example, the OMVs released by intestinal symbiont *Bacteroides thetaiotaomicron* can inhibit the core virulence regulator VirF of *Shigella flexneri* at the post-transcriptional level, thus weakening its ability to invade the colonic epithelium before infection 153.

Under the selection pressure of host immune defense or antibiotic treatment, pathogens will use OMVs to provide protection for the entire microbial community. Take *K. pneumoniae* as an example. When it is exposed to sublethal concentrations of antibiotics (such as polymyxin B), it will induce the production of a large number of OMVs. These Lipid A-rich OMVs act as high-affinity “decoys” that adsorb and neutralize cationic antimicrobial peptides before they reach the bacterial surface 154. The OMVs produced by *M. catarrhalis* can act across species, helping bacterial pathogens such as *Haemophilus influenzae* and *P. aeruginosa*, as well as fungi such as *Candida albicans*, avoid attack by membrane-targeted drugs or serum complement. Through this collaborative defense mechanism, OMVs create a shared protective microenvironment for the entire microbial community, enabling them to survive together during disease progression.

In the recovery phase after infection, the functional focus of OMVs turns to restoring barrier integrity and regulating inflammation. For example, the vesicles derived from the probiotic *E. coli* Nissle 1917 can promote the migration and angiogenesis of endothelial cells, and effectively change the local environment from the state of acute bacterial attack to the state of tissue regeneration and regression. From the above research results, it can be clearly recognized that OMVs are spatiotemporally regulated mediators, which describe the pathobiological trajectory of the interaction between host and microbiota—from defense against invasion, to symbiosis and cooperation, and then to tissue recovery 155.

#### 5.3.2 OMVs in antimicrobial action and antibiotic resistance

**Figure 5:**
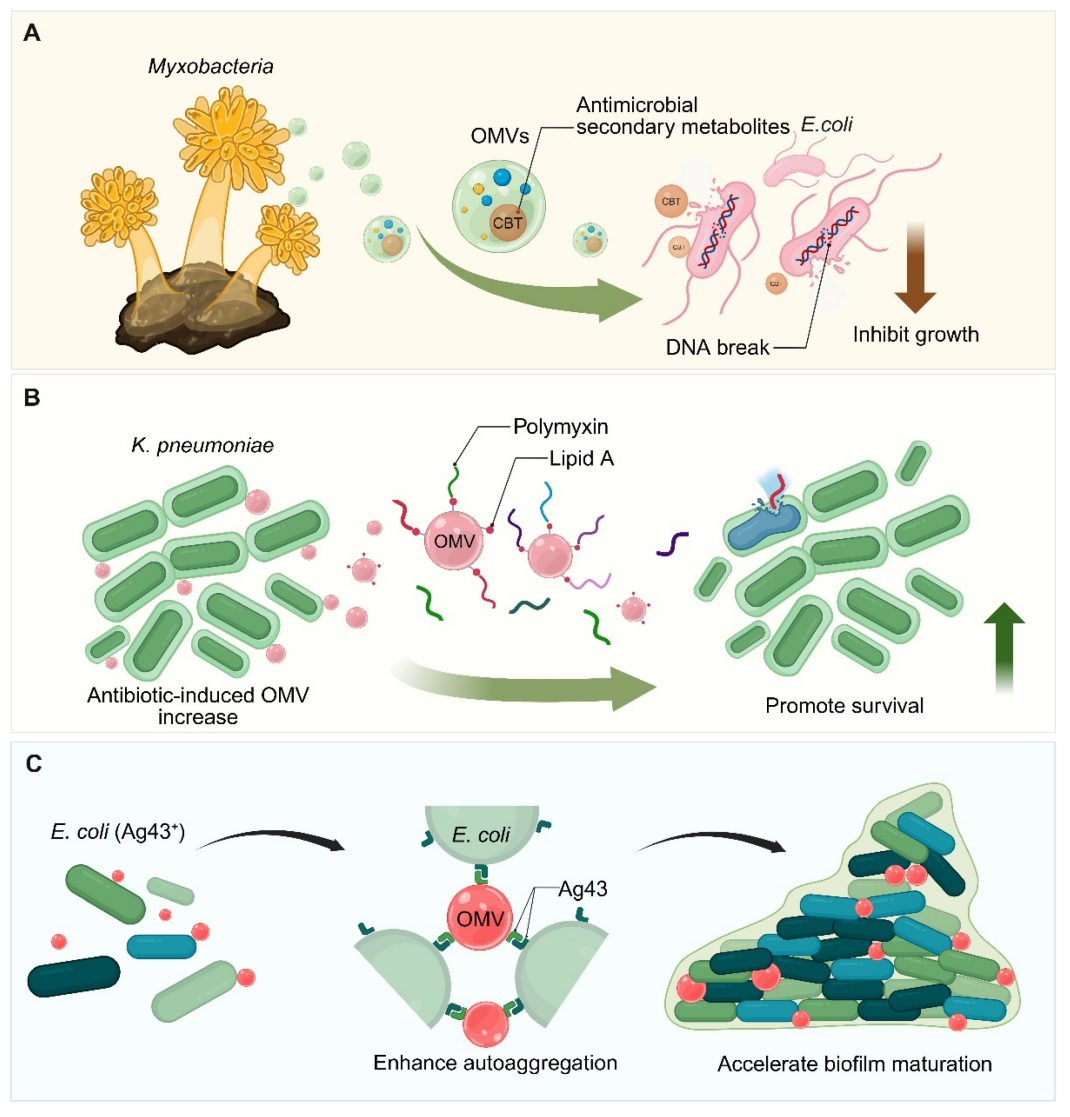
Multifaceted roles of bacterial OMVs in antimicrobial activity and biofilm dynamics. (A) Myxobacterial OMVs intrinsically loaded with antimicrobial secondary metabolites such as cystobactamids target *E. coli* and inhibit bacterial growth. (B) Antibiotic-induced OMV release promotes the survival of *K. pneumoniae* by scavenging polymyxin through lipid A–dependent decoy binding. (C) Ag43-bearing OMVs bind Ag43-positive *E. coli* in an Ag43-dependent manner, thereby enhancing autoaggregation and accelerating biofilm maturation by increasing intercellular contacts and overall biofilm biomass. Ag43: antigen 43; CBT: cystobactamids; OMVs: outer membrane vesicles.

OMVs have become multifunctional regulatory mediators in the fields of microbial competition, host-pathogen interaction and antibiotic resistance. They can directly inhibit bacterial growth by delivering antimicrobial metabolites, enzymes or inducing oxidative stress. For instance, *Myxobacteria*-derived OMVs carrying cystobactamids suppress *E. coli* growth comparably to gentamicin **(Figure 5)**, while *P. aeruginosa* OMVs generate ROS to kill *Streptococcus mutans* (*S. mutans*) 156. Probiotic OMVs also display potent activity, as EcN vesicles suppress methicillin-resistant *S. aureus* (MRSA) 155, and *Ligilactobacillus* EVs inhibit *Salmonella* and *C. jejuni* 157. The hybrid system of *Myxobacteria* OMVs and liposomes modified by bioengineering can realize the targeted delivery of antibiotics to the biofilm of Gram-negative bacteria, while avoiding immune clearance and maintaining the gut microbiome balance 158.

OMVs provide an additional layer of protection at the community level by binding up antimicrobial agents. Lipid A neutralizes cationic peptides such as polymyxins; when under polymyxin B stress, *K. pneumoniae* produces more OMVs to increase cumulative sequestration and cross-species protection **(Figure 5)** 154. They also mediate resistance through enzymatic degradation: OMVs from *K. pneumoniae* (CRKP), MDR *A. baumannii*, or *E. coli* encapsulate carbapenemases (KPC, NDM-1, OXA-97, NDM-5), which degrade meropenem and protect bystander bacteria in vitro and in vivo 159, 160. Also, OMVs enable horizontal gene transfer of antibiotic resistance genes, including plasmid-encoded and chromosomal resistance determinants in *Campylobacter*, and can transiently convey functionally enhanced efflux pumps, such as FE-CmeB, to sensitive strains 161.

In addition to enhancing drug resistance, OMVs can also produce seemingly contradictory synergistic effects with sublethal doses of antibiotics. In *P. aeruginosa*, OMVs can not only adsorb polymyxin B and regulate the expression of drug-resistant genes, but also inhibit quorum sensing at sub-inhibitory concentration of drugs, thereby reducing the virulence, motility and biofilm forming ability of bacteria 162. Using their antigen-presenting properties, the OMVs of *A. baumannii* have been used to isolate human monoclonal antibodies. mAb1416, a monoclonal antibody obtained by immunizing transgenic mice, can specifically recognize KL49 capsular polysaccharide and has a significant preventive protective effect on clinical strains with high mortality in a neonatal sepsis model 163.

To sum up, these findings reveal the dual functional characteristics of OMVs: they can be used not only as carriers for mediating intrinsic antimicrobial activity and population-level drug resistance, but also as an innovative platform for the discovery of therapeutic antibodies and targeted intervention against multidrug-resistant pathogens.

#### 5.3.3 OMV mediated inhibition of pathogen adhesion and virulence

OMVs play a variety of roles in pathogen pathogenesis, especially in the dynamic regulation of biofilm formation and virulence. Studies have shown that OMVs can promote the formation of biofilms. For example, OMVs from *E. coli* are rich in adhesin Ag43, which helps to enhance self-aggregation between bacteria, thus accelerating the maturation of biofilms **(Figure 5)** 164. However, OMVs also possess the ability to disrupt biofilms and inhibit colonization: for example, OMVs of *P. aeruginosa* origin are able to exert anti-biofilm activity against *S. mutans* through a mechanism of membrane damage mediated by ROS 156. OMVs can also reduce the virulence of pathogens by affecting the gene regulatory network. For example, the OMVs of *Bacteroides thetaiotaomicron* can inhibit the expression of VirF in *Shigella dysenteriae*, and then down-regulate the key factors necessary for epithelial cell invasion and intercellular diffusion, thus limiting the development of infection in vivo 153.

Recent studies have revealed that OMVs can mediate a new pathogenic mechanism targeting the host. Through PorV and lipocalin, OMVs secreted by tick *Bacillus burmannii* variant induce a unique cell death mode, "floattosis", in macrophages. This process depends on the endosomal transporter SLC9A9, and amiloride can enhance the host defense by blocking this protein, suggesting that it may become a new therapeutic target 163. In addition, *V. cholerae* OMVs carrying zinc metalloproteinase HapA can internalize into epithelial cells and destroy tight junctions and adhesive junctions. Verification in organ-like and cell sphere models showed that this mechanism could coordinate the degradation of claudin, ZO-1 and β-catenin, thereby enhancing intestinal toxicity 31. On the other hand, carbapenem-resistant *A. baumannii* uses OMVs to deliver the outer membrane protein OMP38 to the host mitochondria, triggering the release of mitochondrial DNA and the activation of cGAS-STING, leading to the excessive production of IFN-β and inflammatory lung injury. It is worth noting that epigallocatechin gallate can inhibit this pathway, showing the potential value of non-antibiotic immunotherapy 165.

In a word, OMVs constitute a multifunctional microbial system: they not only mediate biofilm formation and virulence regulation, but also manipulate host cell function, and participate in the formation of antimicrobial resistance. An in-depth understanding of these mechanisms is essential for the development of next-generation anti-infection strategies and immunotherapies for pathogen–host interactions.

#### 5.3.4 Engineered OMVs for vaccines against bacterial infections

OMVs are widely considered a potential platform for cell-free vaccines because of their inherent adjuvant properties, the ability to present multivalent antigens and the ability to be efficiently ingested by APCs. In terms of clinical application, the OMV vaccines (MenBvac, MeNZB) developed for *N. meningitidis* serogroup B have made key breakthroughs in human immunization, marking important progress in this field 166. Currently, the focus of research is gradually shifting to the use of genetic engineering strategies (e.g., the generalized modules for membrane antigens, GMMA) to enhance the safety, attenuate the endotoxin activity, and enrich the antigenic composition of OMVs 167.

A major focus of OMV vaccine development has been the rational enhancement of immunogenicity and protective efficacy through vesicle engineering. In *A. baumannii*, intranasal OMV immunization effectively reduces airway colonization and prevents systemic dissemination in mouse models 168, while bacteriophage lysin–induced OMVs (LOMVs) offer improved homogeneity, higher protein yield, and lower endotoxin content compared with natural OMVs, yet confer equal or superior protection in pneumonia and bacteremia models 96. For *K. pneumoniae*, glycoengineered GMMA strains generate a bivalent O1/O2 polysaccharide–displaying vaccine with reduced LPS activity 169, and hybrid membrane vesicles (HMVs) that fuse bacterial OMVs with macrophage membrane vesicles and load secreted toxins form multiantigenic nanovaccines (AuNP@HMV@SPs) that markedly diminish inflammatory toxicity and provide full protection in murine septicemia and pneumonia 170. OMV engineering has also been extended to intracellular bacterial pathogens. For example, a triple-gene-deleted *Brucella abortus* strain (Bru-M3) was engineered to remodel its noncanonical LPS, yielding OMVs that induce robust humoral immunity and IFN-γ–producing Th1 CD4⁺ T-cell responses, thereby enhancing bacterial clearance and mitigating tissue damage under both low- and high-dose challenge conditions 171.

In parallel, probiotic chassis have emerged as versatile platforms for scalable and programmable OMV-based vaccines. EcN has been extensively engineered to support high-performance OMV production and cargo delivery. Mutations such as *ΔtolRΔmlaE* markedly increase OMV output, whereas *ΔtolAΔnlpI* enhances heterologous protein secretion, enabling the generation of FI-mOMV nanovaccines that induce robust humoral and cellular immunity against *P. aeruginosa*, as well as TOB-PslG-mOMV nanoantibiotics that synergistically disrupt biofilms 172. Beyond bacterial antigens, engineered EcN has been repurposed as an oral immunization platform in which OMVs mediate systemic translocation of surface-displayed antigens or nanobodies, inducing coordinated mucosal and systemic IgA and IgG responses against diverse pathogens, including viral targets such as SARS-CoV-2 173.

Nanotechnology-driven structural optimization has further improved the stability, targeting efficiency, and immunological potency of OMV vaccines. Coating *Bordetella bronchiseptica* (*B. bronchiseptica*) OMVs onto PEGylated nano–Rehmannia glutinosa polysaccharides yields a stable concentric nanostructure, thereby improving lymph node targeting and DC maturation and ultimately eliciting a robust mixed Th1/Th2/Th17 response and strong antigen-specific protection 174. In a complementary manner, bacterial cellular nanoparticle (CNP) vaccines**,** exemplified by Pa-STING CNPs, are constructed by coating Pa-OMVs onto self-adjuvanted nanocores that activate the STING pathway. This design allows for more efficient APC recruitment to draining lymph nodes and induces a potent adaptive antibody-mediated immune response to protect against lethal pneumonia from hypervirulent or heterologous *P. aeruginosa* strains 175.

Beyond classical vaccination, OMVs are increasingly exploited as multifunctional immunotherapeutic and antimicrobial nanocarriers. Hybrid nanovesicles combining *E. coli* OMVs with CXCR4-overexpressing bone marrow stem cell membranes (mBMSCCXCR4@OMV) exemplify immune-remodeling strategies by homing to the bone marrow niche, reversing local immunosuppression through M1 macrophage polarization, and inducing durable memory B-cell responses to prevent postsurgical relapse in refractory implant-associated infections 176. In multidrug-resistant (MDR) bacterial sepsis, immunomodulatory nanozymes (PdIr@OMVs) deliver nanocatalysts into dysfunctional macrophages, where localized peroxidase-like activity eradicates intracellular pathogens while restoring innate–adaptive immune crosstalk and reversing immunoparalysis 177. In periodontal disease, OMV-encapsulated “nanodisguisers” derived from *F. nucleatum* penetrate pathogenic biofilms, induce oxidative stress via metabolic disruption, and selectively eliminate periodontal pathogens while preserving commensal microbiota 178. Similarly, biomineralized OMVs modified with hydroxamate-type siderophores (SOMV@CaCO₃) provide a pH-responsive system for targeted clearance of both extracellular and intracellular *S. aureus* reservoirs 179.

Finally, OMVs function as adaptable recombinant antigen carriers and immune modulators across diverse infectious contexts. In *H. pylori*, OMVs engineered through deletion of LPS-modifying genes (lpxE, lpxF, futB) and delivery of UreB, VacA, and CagA via the Hbp system elicit a mixed Th1/Th2/Th17 response and reduce bacterial colonization 180; OMVs from nonpathogenic *C. crescentus* offer a low-toxicity platform for antigen presentation 94, whereas BCG-derived OMVs (B-OMVs), which lack LPS, induce TLR2-dependent trained immunity and confer superior protection compared with conventional BCG in polymicrobial sepsis. Mechanistically, B-OMVs reprogram innate immunity through TLR2-driven aerobic glycolysis and epigenetic remodulation, promote hematopoietic stem cell expansion and increased myelopoiesis with a better safety profile 181. Further, OMVs can serve as plug-and-display nanovaccines for the covalent conjugation of heterologous antigens, such as the SARS-CoV-2 receptor-binding domain (RBD), via the SpyTag/SpyCatcher system, which promotes uptake by DCs, activates NOD2 and TLR3 signaling, and generates a strong mucosal IgA response upon intranasal immunization **(Table 2)** 182.

### 5.4 Diagnostic potential of OMVs as biomarkers for infectious diseases

OMVs are emerging as novel biomarkers with great potential for the detection of infectious diseases, owing to their cargo that closely mirrors the molecular composition of their parent bacteria. The unique molecular spectrum of OMVs (including proteins, lipids, nucleic acids, and metabolites) not only reflects the type of parental bacteria, but also accurately represents their biological state, which opens up a new avenue for early diagnosis, accurate pathogen identification, and effective monitoring of disease progression.

#### 5.4.1 OMV mediated pathogen detection and early diagnostic profiling

LPS is a typical structural hallmark of Gram-negative bacteria and is extensively displayed on the surface of OMVs; lipoteichoic acid (LTA) is a characteristic molecular signature of CMVs released by Gram-positive bacteria 184. As a result, dynamic profiling of LPS-positive and/or LTA-positive extracellular vesicles (LPS⁺/LTA⁺ EVs) in plasma is a robust approach to distinguish between bacterial infection and non-bacterial states, and it also helps differentiate between Gram-negative bacteria and Gram-positive bacteria 185. In addition to general bacterial classification, EV-associated markers can also be used for strain differentiation. Notably, penicillin-binding protein 2a (PBP2a) was confirmed to be a major resistance determinant present in EVs released by methicillin-resistant *Staphylococcus aureus* (MRSA) 186. Detection of PBP2a⁺ EVs thus represents an extremely promising plasma biomarker for the early detection of MRSA-associated antibiotic resistance 186.

The diagnostic potential of OMVs is particularly evident in enabling early detection and longitudinal monitoring of infections in complex clinical settings 187. In a male mouse pneumonia model, a polymyxin B–fluorescein (PmBF) probe targeting LPS was used to selectively label the circulating OMVs. The results showed that the number of PmBF⁺ OMVs increased significantly as early as 6 h after infection 188. It is worth noting that this increase occurred before blood cultures turned positive, highlighting the great potential of OMVs as biomarkers of early bacterial infection. In addition to early detection, PmBF⁺ EVs also show obvious advantages in differential diagnosis, effectively distinguishing bacterial infection from non-bacterial diseases (including viral and mycoplasma infections) 188.

#### 5.4.2 OMV based infection monitoring and therapeutic assessment

In severe infections such as sepsis, empirical broad-spectrum antibiotic therapy often leads to negative blood culture results, which complicates pathogen identification. However, because bacteria continuously release outer membrane vesicles—a process that may be further enhanced under antibiotic pressure - detection of these vesicles provides a key alternative molecular pathway for pathogen identification. Therefore, this method has particular value for the molecular diagnosis of Gram-negative bacterial sepsis 189.

In addition, dynamic analysis of the abundance of OMVs can be used as a reliable marker of disease progression and treatment response 186. For example, after antibiotic treatment, the proportion of PBP2a⁺ EVs in plasma gradually decreases, which provides a precise quantitative indicator for monitoring treatment efficacy 186. In local infectious diseases, such as periodontitis, we observed an increased level of salivary LPS⁺ OMVs, further confirming its potential as a noninvasive diagnostic biomarker 187. OMVs can not only be used as static diagnostic markers, but also as dynamic biological indicators, closely linking infection monitoring and real-time evaluation of therapeutic intervention.

The therapeutic assessment of complex diseases is increasingly carried out through dynamic monitoring of OMV abundance as well as cargo composition. In metabolic disease research, delivery platforms based on OMVs have been used to quantitatively assess detoxification efficacy in vivo. OMVs carrying specific enzymes (e.g. uricase) can be generated by engineering the type zero secretion system of bacteria, and these vesicles are able to cross the intestinal barrier to enter the circulation and continuously catalyze the degradation of metabolites, such as uric acid, enabling quantitative assessment of metabolic clearance 190. In inflammatory disease models, efficacy can be assessed by tracking the bioactivity of therapeutic vesicles. For example, the engineered EcN was able to selectively encapsulate PD-L1-mFc protein fragments in OMVs, enabling quantitative assessment of mucosal repair and restoration of intestinal homeostasis in autoimmune disease models 191. Similarly, oral micro- to nano-scale genome-editing systems use lipopolysaccharide-deleted OMVs to deliver CRISPR–Cas9 targeting TNF-α in macrophages, providing a molecular endpoint for gene therapy efficacy 192.

#### 5.4.3 Challenges and solutions in the clinical translation of OMV based biomarkers

Although OMVs, as potential diagnostic biomarkers, have shown broad application prospects, there are still many key obstacles in the process of their clinical translation. OMVs show obvious diversity in biophysical and biochemical characteristics, which is mainly determined by the diversity of strains and external environmental conditions, and is reflected in changes in morphology, size, and molecular composition. At the same time, it is still a challenging task to efficiently isolate and purify these vesicles from complex biological samples. These factors together constitute the main bottleneck in the current development of this field 184, 185, 189. In addition, the scientific community has not yet reached an agreement on which markers can generally represent bEVs, which also makes the standardization of related detection and characterization more complex and difficult 184.

In order to overcome these limitations, current research is gradually focusing on the development of analytical techniques with higher sensitivity and specificity. Among them, nano-flow cytometry combined with high-specific molecular probes such as PmBF or a targeted PBP2a antibody can realize the quantitative analysis of OMVs at the single-particle level, so as to effectively distinguish bEVs from the host extracellular vesicles 186, 188, 193. At the same time, the development of new biological recognition materials (such as LPS molecularly imprinted polymers) provides a feasible way to capture OMVs with high selectivity and efficiency, and lays a foundation for subsequent proteomics research and the construction of an early bacterial infection diagnosis platform 194.

Looking forward to the future, the construction of a standardized and repeatable OMV separation and characterization system is of key significance for improving data comparability and promoting the rapid translation of vesicle biomarkers with great potential for clinical application.

## 6. Summary and perspectives

### 6.1 Key advantages of OMVs

OMVs, as naturally occurring, non-replicating biological nanoparticles, have both antigen delivery and endogenous adjuvant activities, and can synergistically activate innate and adaptive immune responses. With their unique structure and function, OMVs have become an efficient biological nanoplatform with great potential in the cross-fields of microbiology, immunology, and nanomedicine. Their nanoscale structure, rich molecular cargo, and biomimetic membrane composition confer multiple inherent advantages on OMVs, including efficient uptake by APCs, lymph node tropism, natural conformational multivalent antigen presentation, and the ability to stimulate balanced humoral and cellular immune responses 195. In addition, the OMVs show excellent versatility in cargo carrying (encompassing proteins, nucleic acids, small-molecule drugs, and gene editing systems), and have excellent structural stability under different physical and chemical conditions, supporting large-scale production and flexible drug delivery routes 150, 196. These characteristics collectively establish OMVs as one of the most promising new-generation platforms in the fields of vaccines, cancer immunotherapy, antimicrobial therapy, and beyond.

### 6.2 Translational challenges and safety considerations of OMVs

Although engineered OMVs have shown great potential in the diagnosis and treatment of diseases, a series of safety issues still need to be fully evaluated in order to truly realize clinical transformation. Among them, a key problem that cannot be ignored is the narrow toxicity window of OMVs, which is closely related to the rich LPS and other PAMPs on the surface of OMVs 93. On the one hand, these ingredients give OMVs strong immune stimulation ability, on the other hand, when the dosage is not controlled properly, they may also cause excessive systemic inflammatory response 90. In vivo studies have shown that this excessive immune activation may further lead to serious adverse reactions, such as sepsis, acute organ injury, liver inflammatory cell infiltration, pulmonary fibrosis and splenomegaly. Therefore, in the treatment strategy design based on OMVs, how to maintain the safety within an acceptable range while ensuring sufficient immune activation effect is still a core problem that needs to be weighed and solved 93, 197. In addition, because OMVs can act on multiple pattern recognition receptors at the same time, we need to pay attention to whether it will cause excessive immune activation and whether it may break the original immune tolerance state of the body 198, 199. Especially in the case of repeated administration or long-term administration, this problem deserves more attention. On the other hand, the content carried by OMVs is complex, which further increases the difficulty of evaluation. For example, some components derived from pathogens, such as VacA of *H. pylori* and opacity-associated proteins of *N. meningitidis*, may have immunomodulatory effects, thus affecting the therapeutic effect 198. This problem is particularly critical in some specific application scenarios, such as tumor immunotherapy, because such treatment usually requires continuous and effective T cell activation, and any factors that may interfere with this process may further affect the final treatment performance.

Corresponding to the safety issue, whether OMVs can achieve large-scale clinical application also depends on whether a series of manufacturing problems can be effectively solved. Firstly, the yield of OMVs produced by natural strains is usually low, which not only limits its large-scale supply capacity, but also increases the complexity of the production process to a certain extent. In addition, the biogenesis of OMVs is a dynamic process regulated by metabolism, and is very sensitive to culture conditions, such as environmental stress, oxygen supply, nutritional status, the growth stage of bacteria and the composition of culture medium 198. In this case, differences in vesicle particle size, molecular composition, protein profile and LPS content often occur between different batches, which may further affect the efficacy and safety. At present, OMVs still lack a set of fully standardized and high-purity technical processes in terms of separation, purification and characterization, which also makes it face great challenges in meeting the consistency and quality control standards required by regulatory approval and current Good Manufacturing Practice production 86. However, these problems do not mean that the OMV platform lacks transformation prospects, but that the promotion of its clinical application still depends on a more controllable engineering transformation strategy, a more stable and reliable production process, and a more standardized and unified quality evaluation system. Only by further improving these key links can we better promote the OMV-related platform to move toward future clinical translation.

### 6.3 Recommendations for advancing OMV engineering

From the perspective of translational medicine, to fully unleash the clinical potential of OMVs, it is necessary to break through fragmented engineering improvement ideas and establish a more coherent, application-oriented design concept. Future OMV development should be guided by clear principles to match and synergistically enhance its biological performance with clinical feasibility. The future research and development of OMVs should no longer passively carry out local optimization or gradual repair, but should be based on the unified core principle, and strengthen the clinical feasibility while taking into account the biological performance.

The core consideration in this process is safety, which must be incorporated into the overall planning at the initial stage of the design of the OMVs. Only when the strategies of lipid A structural remodeling, proteomic refinement, and the selection of non-pathogenic or endotoxin-free bacteria as the chassis are implemented as basic design principles, can it maximize its efficacy, avoid excessive inflammatory reaction and achieve more balanced immunoregulatory characteristics while ensuring the therapeutic effect 94, 95.

The OMV platform also needs to be able to adapt to different clinical contexts. The value of surface and structural engineering strategies (such as polymer shielding, supramolecular coating and membrane fusion technology) does not lie in the complexity of the technology itself, but in their ability to make OMVs have controllable biodistribution, immune activation capacity, and higher in vivo tolerance under the guidance of clear pharmacological and immunological goals 103, 122.

As the vaccine and treatment platform based on OMVs gradually moves toward clinical application, its large-scale capacity and robustness of production process have become indispensable key considerations in biological design. The establishment of standardized high-throughput production and purification processes, combined with the strategy of improving production and strict quality control system, is of decisive significance to ensure the consistency between batches and realize clinical transformation 96. A modular antigen display system, which can decouple the antigen presentation process and gene operation, provides a feasible technical path for rapid iteration, multivalent preparation development and personalized vaccine design.

Finally, whether OMVs can be continuously promoted in the clinic largely depends on whether the regulatory system and the standardization framework can achieve effective convergence in the early stage. The establishment of a unified reporting standard, reliable efficacy testing methods, and consensus evaluation criteria for the characteristics of OMVs are crucial to ensure the comparability between different studies, promote regulatory evaluation, and ultimately promote clinical application. These considerations highlight an important idea: OMV engineering should not be regarded as an isolated technological attempt, but as a multidisciplinary transformation process through design, production and supervision.

### 6.4 Future directions toward function oriented classification and rational design of OMVs

Research on OMVs is gradually shifting from relying solely on technological breakthroughs to building a more biologically adaptive integrated system, but there is still a significant gap between mechanistic research and clinical translation. To better bridge this gap, we may attempt to adjust the existing approach by no longer classifying them primarily based on their source strains or biological processes, but instead by emphasizing their potential functions within host cells and their predictability. Therefore, we propose a function-oriented classification method for OMVs, which changes from "source-driven" to "function-driven", and classifies them according to the potential role of OMVs after entering the host, including: (i) Metabolic-Reprogramming (MP) OMVs, which may change the metabolic state and adaptation strategy of cells by regulating the intracellular metabolic network and stress response pathway; (ii) Immune-Modulating OMVs, which are characterized by the ability to activate pattern recognition receptors in a specific way, thus shaping the direction and intensity of innate immune response; (iii) Organelle-Targeting OMVs; this type of OMVs has the potential to accurately deliver bioactive molecules to specific subcellular structures, so that its role has a clear spatial location; (iv) Barrier-Interacting OMVs; such OMVs may be optimized to enhance the ability to cross complex biological barriers, so as to achieve more efficient cross-tissue transport. Based on this perspective, it can be further imagined that through rational design of lipid composition, protein structure motifs and surface modification modes, OMVs are expected to show greater programmability and predictability at the genetic, epigenetic and metabolic levels, so as to expand their application from the traditional immune regulation to more extensive and refined biomedical fields.

At the same time, the rapid development of spatial multiomics and single-cell technology enables us to better characterize how OMVs behave within diverse tissue microenvironments and how they interact with surrounding cells. These emerging findings are also gradually promoting a more biologically realistic design concept of “microenvironment-responsive OMVs”. The focus is no longer limited to optimizing the intrinsic composition of OMVs themselves, but more consideration is given to the performance in a specific local environment, such as the state of surrounding cells, the strength of local immune response and the variations in metabolic conditions. In the future, relevant research may use more data-driven and even AI-assisted design strategies to integrate multidimensional data such as OMVs lipidomics, host transcriptome information and immune spectrum, so as to continuously optimize the composition and functional performance of OMVs. In the long run, this line of thinking will not only support more tailored optimization of OMVs for different disease contexts, but may also enable the development of predictive models for their efficacy and safety. At the same time, the concept of quality control may also change: from the concept of end-point testing to greater emphasis on process. For example, the dynamic monitoring of the biogenesis process of OMVs and the continuous evaluation of the stability of their composition will further enhance the reliability of OMV clinical translation in the future.

In the future, OMVs are more likely to be used as a "collaborative tool" for combination therapy than as a single therapy. From the perspective of translational research, it is suggested to promote this in the following aspects: establish recognized reference standards for lipidomics and proteomics to facilitate comparison of data between different studies and solve the current problems of inconsistent standards and difficult horizontal comparison; Secondly, it is necessary to establish a more systematic and standardized preclinical evaluation system to comprehensively evaluate key indicators such as biodistribution, immunogenicity and long-term safety, which will directly affect whether it can be successfully promoted to the clinical stage. At the same time, in early-stage clinical studies, it is suggested to incorporate molecular typing information and biomarkers into the trial design, so as to more accurately identify patients who really benefit and achieve individualized matching. In this broader context, OMVs may no longer serve merely as standalone therapeutic tools, but gradually play the role of "coordinator", organically integrating various means such as immunotherapy, chemotherapy, physical therapy and image guidance, so as to improve the effect of combined treatment strategy as a whole.

## Figures and Tables

**Table 1:** Overview of currently available OMV engineering strategies for cancer therapy.

Model of cancer	OMVs from	Engineering type	Payloads	Functions	Reference
GBM	*E. coli* BL21 (DE3)	Genetic modification co-expressing surface peptides CPP and CLT	Ferroptosis inducer RSL-3	Crosses BBB; targets GBM; enhances CD8+ T-cell infiltration; induces IFN-γ-mediated ferroptosis	43
Melanoma Breast cancer	*E. coli* BL21	Ampicillin; PDA coating; ICG loading	CAT; DNA; ICG photosensitizer	Reprogram TME; relieve hypoxia; cGAS-STING activation; ICD; boost CD8^+^ T cells	92
Colon cancer	*Salmonella* YB1	OMV-Wrapped nanoenzyme nanoreactor	Oxaliplatin; MnO₂ nanoenzyme	Relieve hypoxia; activate cGAS-STING; enhance metalloimmunotherapy	99
TNBC PADC	*E. coli* BL21 (DE3)	Biomimetic fusion membrane with hyaluronidase decoration and PD-L1 genetic knockout	Oxaliplatin; Gemcitabine; nucleic acids	Enables neutrophil hitchhiking; degrades ECM; ensures homologous targeting; activates antitumor immunity	100
Breast cancer Colon cancer Melanoma	*E. coli* BL21 (DE3)	Mesoporous silica nanoparticles cloaked with OMVs forming a modular nanohybrid platform	Immunostimulatory cytosine-phosphate-guanine oligodeoxynucleotides (CpG-ODNs)	Restores T-cell metabolic fitness; enhances mitochondrial oxidative phosphorylation; reshapes tumor immunity	105
Colon cancer	*E. coli* BL21 (DE3)	Genetic surface display (ClyA); MMP-2-responsive peptide; surface calcium phosphate biomineralization	IRG1 inhibitor (IRG1-IN-1) and matrix metalloproteinase-2-responsive PD-L1 nanobody	Inhibits itaconic acid; restores chemokine secretion; enhances T-cell infiltration; blocks PD-L1	106
Breast cancer Melanoma	EcN	Biomineralized OMVs with FA-decorated manganese-doped calcium phosphate nanoshell	LOX, Ca^2+^, and Mn^2+^ ions	Activate cGAS-STING, deplete lactate, reprogram TME, and boost antitumor immunity	107
Colon cancer	*E. coli* MG1655	Genetic surface-display of nanobodies and chemical loading of photosensitizers	CDH17-targeting nanobody (Nb289) and IRDye700DX photosensitizer	Activates cGAS-STING pathway; induces ICD; enhances photoimmunotherapy and checkpoint blockade	109
Breast cancer	*E. coli* BL21	Chimeric nanozyme-armed OMVs via Da-Fe metal-phenolic network and in situ AuNP growth	Ultrasmall gold nanoparticles, ferric ions; dopamine-functionalized PEG	Induces glucose starvation; chemodynamic therapy; ferroptosis; ICD; synergistic TME immune reprogramming	110
Hepatocellular carcinoma Melanoma	*E. coli* MG1655	Macrophages carrying C16-ceramide-fused drug-loaded OMVs	R848 and INCB024360	Target tumors; specific phagocytosis; remodel TME; activate immunity	111
Breast cancer	*E. coli* BL21	Optogenetic bacterial platform secreting therapeutic OMVs	GM-CSF and siRNA targeting SIRPα	Reprogram TAMs (M2 to M1); block CD47-SIRPα; inhibit tumors	112
Breast cancer	EcN	OMV-coated L-arginine nanomicelles integrated into adoptive macrophages	L-arginine and arginase inhibitor L-norvaline	Immunometabolic engineering; activate M1 phenotype; restore nutrient availability	113
Melanoma	*E. coli* DH5α	OMV-cancer cell hybrid membrane-coated hollow polydopamine nanoparticles	Bacterial antigens and hollow polydopamine (photothermal agent)	Homotypic targeting; photothermal therapy; activate DCs; eradicate tumors	114
Hepatocellular carcinoma Breast cancer Colon cancer	*Salmonella*	Fusion of OMVs and PTSLs; cypate incorporation	CD38 siRNA (modified with anti-CD7-9R); cypate; anti-PD-1 combination	Cascade targeting; PTT effect; CD38 knockdown; T cell cytotoxicity/proliferation	115
Breast cancer	*E. coli*	Surface cloaking with hafnium-phenolic networks via metal-polyphenol coordination	Hafnium ions (Hf^4+^), TA, and endogenous catalase	Hypoxia relief; radiosensitization; tumor antigen release; DC maturation; systemic radioimmunotherapy	116
Hepatocellular Carcinoma Colorectal cancer	*E. coli* BL21	Metal-phenolic network coated OMV-encapsulated elesclomol actuator	Elesclomol, Cu^2+^, and Tannic acid	Induce CPApoptosis; ICD release; reprogram TME; adaptive immunity	117
Melanoma Colon cancer	*E. coli*	STING agonist-loaded synthetic bacterial vesicles	STING agonist (DMXAA)	Synergistic DC activation; enhance IFN-β; increase T cell infiltration	118
Breast cancer	*E. coli* MG1655	Genetic aCD47 display; Ce6 membrane loading; neutrophil-mediated hitchhiking delivery	CD47 antibodies; photosensitizer chlorin e6	Precise postoperative targeting; macrophage reprogramming; CD47 blockade; light-triggered antitumor immunity	119
PCa	EcN	Encapsulation of oncolytic virus via the membrane extrusion method	Genetically engineered oncolytic virus OH2	Remodels TAMs (M2 to M1); induces direct oncolysis; activates systemic antitumor immunity	120
Bladder cancer Breast cancer Melanoma	*E. coli* BL21	CRISPR/dCas9-ClyA fusion gene packaging	CXCL9; IL-12-encoding genes	Enhance T cell chemotaxis/activation; synergize ICB therapy	121
Melanoma Breast cancer	*Salmonella*	Fusion of melanoma CMVs and Salmonella OMVs; ICG/PLGA core (PI@EPV)	Melanoma antigens (gp100, TRP2); ICG/PLGA photothermal core	Synergistic PTT/immunotherapy; robust DC activation; CTL immunity; inhibits tumor growth/metastasis	122
Melanoma	*E. coli*	Genetic pDNA expression; encapsulated in iontophoresis-driven MN array	gp100 antigen; CCL21 chemokine	TCI/vaccine; enhance DC migration/maturation; boost CTL cellular immunity	123
Breast cancer	*E. coli* BL21 (DE3)	PD-1-displayed OMV-coated zeolitic imidazolate framework (ZIF-8)	MicroRNA miR-34a	Targeted miRNA delivery; pH-responsive release; PD-1/PD-L1 checkpoint inhibition	125
Melanoma	*E. coli* DH5α	Inhaled glycine-induced outer membrane vesicles (Gomv) vaccines	Immunogenic OmpA/OmpC and reduced LPS	Alveolar macrophage activation; M1 polarization; antigen release; CTL activation	127
Melanoma Breast cancer	*R. palustris*	Maleimide surface modification for antigen capture	PTT-released neoantigens/DAMPs captured	Antigen transport to TDLNs/margin; boost CTLs, inhibit metastasis	128
Colon cancer	EcN	Biomimetic hybrid vesicles formed by membrane extrusion of OMVs and cellular vesicles	NIR-II fluorescence; membrane probes (BMP1) and anti-PD-L1 scFv	NIR-II imaging; targeted photothermal therapy; PD-L1 blockade; M2-to-M1 macrophage repolarization	129
Breast cancer	*E. coli* DH5α	OMV-camouflaged PLGA nanoparticles (OMV@PCB NPs)	Photosensitizer (Ce6); TGF-β inhibitor	PDT-assisted neutrophil hitchhiking; N1 polarization; ELANE release; TME reprogramming	130
Breast cancer	*E. coli* BL21	Genetic HAase surface display and chemical PBA conjugation	Cholesterol-modified CD73 siRNA	Degrades ECM; alleviates hypoxia; silences CD73; activates systemic antitumor immunity	131
Bladder tumor	*E. coli* BL21	Magnetic macrophage microrobots loaded with bioengineered OMVs	Hirudin and mastoparan 1 anticancer peptides	Magnetic propulsion; tumor targeting; multimodal therapy; immune regulation	132
Colorectal cancer Colon adenocarcinoma Melanoma	*E. coli* DH5α	OMV-loaded Mg-based micromotors with Au, TiO2, PLGA, and chitosan coatings	Immunostimulatory bacteria-derived OMVs	Mechanically disrupt tumors; induce cell death; recruit immune cells; and activate systemic immunity	133
GBM	*ΔmsbB E. coli*	Angiopep-2 targeted; ROS-responsive; attenuated OMV-shell	siCD47; doxorubicin	Overcome dual immune resistance; reprogram macrophages; robust immunity	134
Breast cancer	EcN	In vivo "cell factories" for self-assembled RNA-loaded OMVs	PD-L1 siRNA	In situ RNA delivery; PD-L1 silencing; activate antitumor immunity	135
TNBC	*ΔmsbB E. coli*	pH-responsive; charge-reversal; dual-targeted NPs	PTX; Arg-1 siRNA	Reshape TME; repolarize TAMs; activate T cells	136
PDAC	EcN	NP41-modified OMV system loading larotrectinib	Larotrectinib; NP41	Target nerves; repolarize M2 TAMs; disrupt nerve-cancer crosstalk; augment chemotherapy	137
Colon cancer	Engineered *E. coli* Rosetta	Surface display of Lyp1-Traptavidin fusion protein	Biotinylated anti-PD-L1 antibody and M6P	Targeted lysosomal degradation; unleash anti-tumor immunity	138
Breast cancer	*B. fragilis*	pH-responsive MnO_2_ nanocloaking via biomineralization	MnO_2_ adjuvant; Anti-PD-L1 combination	Reduce toxicity; activate cGAS-STING; boost DC maturation; synergizes anti-PD-L1	139
Melanoma Breast cancer Colon cancer	*A. muciniphila*	Hybrid fusion of cationic liposome	PD-L1 trap plasmid	Synergistic PD-L1 blockade; promote DC/CTL activation; reverse CTL exhaustion	140
Melanoma Colon cancer	*E. coli* MG1655	Maleimide surface modification	MerTK inhibitor UNC2025; tumor-associated antigens	MerTK blockade; TAA capture; LN transfer; boost CD8^+^ T immunity	141
Melanoma Colon cancer Breast carcinoma	*S. typhimurium* VNP20009	Coating *Salmonella* with NP^+^ cationic polymer nanoparticles	Captured tumor/neoantigens; DAMPs; *Salmonella* flagellin adjuvant; αPD-L1 combination	Active transport of antigens to tumor margin; DC/T cell activation; systemic antitumor/abscopal effect; inhibits metastasis	142
HPV-positive TC-1 tumor	*Salmonella* SL7207	Poly-L-arginine E7 peptide-anchored	HPV16 E7 antigen peptide	Activate E7-specific CD8^+^ T cells; boost adaptive immunity; suppresses tumors	143
Melanoma	*E. coli* Rosetta (DE3)	Genetic ClyA-SPAb/ClyA-SpyC; αDEC205 antibody binding (OMV-DEC)	SpyTag-labeled antigens; LPS adjuvant; αPD-1/Ibrutinib combination	Overcomes MUO; persistent DC uptake; strong CTL activation; inhibits metastasis; reduces MDSCs	144
Melanoma Colorectal cancer	*E. coli* Rosetta (DE3)	Genetic fusion of ClyA with SpC/SnC catchers	OVA; TRP2; ADPGK neoantigens	LN accumulation; enhances DC presentation/maturation; boosts CTLs; inhibits metastasis; induces memory	145
Melanoma Colon cancer	*E. coli*	Lysozyme; sonication; high pH treatment	Tumour tEV; OMP adjuvant; combined with αPD-1 antibody	Safe adjuvant; Th1/CTL immunity; DC activation; inhibits growth/metastasis; αPD-1 synergy	146
Pancreatic Colon cancer Melanoma	*E. coli* Rosetta (DE3)	Genetic SpyC/SPAb fusion; αPD-L1 antibody surface modification	Antigen (SpyT-OVA peptide); αPD-L1 antibody	Modulates second signals; enhances DC uptake/presentation; boosts CTL immunity; inhibits metastasis/recurrence	147
Colorectal cancer Melanoma	*E. coli* Rosetta (DE3)	SIRPα-Fc fusion; CaP coating; GM-CSF loading	GM-CSF; SIRPα-Fc protein (CD47 inhibitor)	Induces TIrV; targets/modulates TAMs; M1-like phagocytosis; boost T cell response	148
Metastatic melanoma model (lung metastasis)	*E. coli* DH5α	Electrostatic absorption; R4TRP2 peptide; tunable surface charge	R4TRP2 peptide antigen (SVYDFFVWL)	Elicits innate/adaptive immunity; enhances CTLs; inhibits metastasis; prolongs survival	149
Melanoma Colon adenocarcinoma	*E. coli BL21* (DE3)	Genetic fusion of L7Ae/LLO to ClyA; plug-and-display strategy	Box C/D sequence-labeled mRNA antigens (OVA/ADPGK)	Rapid mRNA display; DC uptake/presentation; strong antitumor T cell response; inhibits metastasis; long-term memory	150
Colon cancer	*E. coli* BL21 (DE3)	Mal-PEG4-NHS surface conjugation; IDO inhibitor 1-MT loading via electroporation	IDO inhibitor 1-MT; captured PTT-released tumor antigens	Antigen capture; reverses IDO suppression; boosts DC maturation/CTLs; systemic effect/memory	151
TNBC	*A. muciniphila* and engineered* E. coli*	Hybridization, surface display of targeting peptide, and electroporation-based drug loading	Tetrathiomolybdate	Copper depletion; metabolic reprogramming (OXPHOS to glycolysis); immune restoration; metastasis inhibition	152

1-MT: 1-methyl-tryptophan; CaP: calcium phosphate; cGAS: cyclic GMP-AMP synthase; CAT: catalase; CTL: cytotoxic T lymphocyte; CXCL9: CXC chemokine ligand 9; DAMPs: damage-associated molecular patterns; DC: dendritic cell; EcN: *E. coli* Nissle 1917; EPV: eukaryotic-prokaryotic vesicle; GBM: glioblastoma; GM-CSF: granulocyte-macrophage colony-stimulating factor; ICB: immune checkpoint blockade; ICG: indocyanine green; IDO: indoleamine 2,3-dioxygenase; LN: lymph node; LLO: listeriolysin O; LOX: lactate oxidase; LPS: lipopolysaccharide; MDSC: myeloid-derived suppressor cells; MerTK: myeloid-epithelial-reproductive tyrosine kinase; MUO: maturation-induced uptake obstruction; NP41: nerve-binding peptide 41; OVA: ovalbumin; PADC: pancreatic ductal carcinoma; PAMPs: pathogen-associated molecular patterns; pDNA: plasmid DNA; PDAC: pancreatic ductal adenocarcinoma; PDT: photodynamic therapy; PI: poly(lactic-co-glycolic acid)-indocyanine green moiety; PLGA: poly(lactic-co-glycolic acid); PTSLs: photosensitive thermosensitive liposomes; PTT: photothermal therapy; PTX: paclitaxel; R4TRP2: positively charged antigen (RRRRSVYDFFVWL); SIRPα: signal regulatory protein-α; SPAb: domain B of staphylococcal protein A; SpyC: SpyCatcher; STING: stimulator of interferon genes; TAA: tumor-associated antigen; TAMs: tumor-associated macrophages; TCI: transcutaneous immunization; TDLNs: tumor-draining lymph nodes; TIrV: trained immunity-related vaccines; TME: tumor microenvironment; TNBC: triple-negative breast cancer; TRP2: tyrosinase-related protein 2; αPD-L1: anti-programmed death-ligand 1 antibodies; Δ means gene deletion.

**Table 2:** Overview of OMVs as a bacterial vaccine platform.

Targeted pathogen	OMVs-producing bacteria	Type of OMVs	Antigen (and location)	Display system	Stage of development	Model	Route of administration	Adjuvant used	Safety/Toxicity	Key Efficacy Findings	Reference	

Platform Vaccine (not targeting the host bacterium)	Engineered *C. crescentus* CVOM-2(*ompA2R351A ΔrsaA* mutant)	b-OMVs	Recombinant protein mCherry; Vesicular lumen	OMV	Preclinical	BALB/c mice	IP: 45 µg OMV, 2 doses, 2-week interval.	Self-adjuvanticity	Significantly improved safety: attenuated innate immune activation (no IL-1β; reduced TNF-α) and minimal discomfort in mice; due to structurally distinct LPS.	OMV-mCherry: induced adaptive immunity. CVOM-2: 7-fold higher antigen loading; 4-fold higher OMV yield vs. benchmarks.	94	
*A. baumannii*	*A. baumannii* WHG40137	LOMVs	Complex mixture of bacteria-derived antigens	LOMVs	Preclinical	BALB/c mice	IM: 10 µg, 3 doses, 2-week interval; IN: 10 µg, 3 doses, 2-week interval or single dose.	IM: Imject Alum; IN: Self-adjuvanticity	LOMVs exhibited minimal cytotoxicity; lower endotoxin; and improved homogeneity vs. nOMVs.	LOMVs: potent; broad protection vs. systemic & pulmonary challenge; IM effective; single IN superior; stronger mucosal cytokine response for enhanced pneumonia protection.	96	
Gram-negative pathogens	*M. xanthus* SBSr 073 and *C. ferrugineus* 23	b-OMVs	Ciprofloxacin encapsulated within the vesicle/hybrid lumen	Biomimetic hybridization	Preclinical	C57BL/6JRj mice	Oral (Gavage): 200 µL (1.0 × 10^12^ particles/mL); 7 doses, every other day for 2 weeks	Self-adjuvanticity	Evades immune clearance; high biocompatibility; maintains host gut microbiome balance	Superior biofilm penetration; enhanced intracellular delivery via hybrids; prolonged extracellular drug exposure	158	
*S. enterica* serovar Enteritidis *S. enterica* serovar Typhimurium	Engineered *S. enterica* serovar Enteritidis and* S. enterica* serovar *Typhimurium* strains (*tolR*, *msbB*, and *pagP* mutants)	b-OMVs	OAg component of LPS; surface	GMMA	Phase Ⅰ	Healthy human adults aged 18–55	IM: OAg 10.6 µg or 40 µg; 3 doses (M0, M2, M6).	Alhydrogel (0.35 mg Al^3+^ / 0.5 mL dose)	No major safety concerns; with only mild; localized adverse events; genetic detoxification (*ΔmsbB/ΔpagP*) effectively reduced reactogenicity.	Vaccine: potent; durable antibody responses; high IgG & SBA titers sustained 1 year; broad activity vs. heterologous STm strains.	167	
*A. baumannii*	*A. baumannii* ATCC 17978*A. baumannii* BAL_084*A. baumannii* BAL_276	WT-OMVs	LPS and cell surface proteins, surface	OMV	Preclinical	Specific pathogen-free BALB/c mice	IM/SC: 100 FU in 50 µL; IN: 500 FU in 100 µL.	Self-adjuvanticity	Low systemic inflammation, with reduced pro-inflammatory cytokines IL-6 and minimal weight loss.	IN: induced respiratory IgA, blocked systemic spread. SC: superior to IM; reduced pulmonary & systemic bacterial loads.	168	
*K. pneumoniae*	Engineered* E. coli* W3110 *ΔwbbH-LΔlpxM::lpxE*	b-OMVs	O-polysaccharide (OPS); surface	geGMMA	Preclinical	BALB/c mice	SC: 12.5 µg OPS; 3 doses (D0, D14, D28).	Self-adjuvanticity	Excellent safety: Attenuated lipid A; stable vitals; low systemic inflammation; normal serum biochemistry.	Bivalent vaccine: >1000-fold IgG increase; balanced Th1/Th2; complete protection vs. *K. pneumoniae* sepsis with near-sterile organs.	169	
*P. aeruginosa*	Clinically isolated *P. aeruginosa* strain PA17ZC010	WT-OMVs	Multiantigenic system; surface	HMVs	Preclinical	ICR mice	SC: 2 µg OMV; 3 doses (D0, D7, D14).	Gold Nanoparticles	Highly favorable safety profile; with reduced toxicity versus controls; normal physiological parameters; no tissue injury; and high blood/cytocompatibility.	Vaccine: 100% survival in lethal septicemia; near-sterile organs; robust humoral/cellular immunity; enhanced DC/B cell activation; controlled hyperinflammation.	170	
*B. abortus*	*B. abortus*	b-OMVs	Remodeled noncanonical LPS; surface	Genetic remodeling	Preclinical	BALB/c mice	SC: 30 µg OMV; 2 doses (D0, D21)	Endogenous remodeled LPS	Transient 5-10% weight loss	Robust humoral/Th1 response; effective bacterial clearance	171	
*P. aeruginosa*	EcN	b-OMVs	OprF190-342-OprI21-83 fusion protein (FI); surface	ClyA-fusion protein system (ClyA-FI-His6)	Preclinical	BALB/c mice	SC: 10 µg per dose on Days 0, 14, and 28	Self-adjuvanticity	Reduced endotoxin activity via diacylation; excellent biocompatibility; no significant toxicity or weight loss in mice	Induced high IgG titers (2.4 × 10^5^); 83.3% survival in lethal challenge; 2142.9-fold lung bacterial load reduction	172	
SARS-CoV-2	EcN	b-OMVs	Spike-RBD or anti-spike nanobodies	Intimin or Lpp-OmpA anchors	Preclinical	C57BL/6J mice	Oral: ~1×10^7^ CFU; 4 doses (D0, D7, D14, D21)	Endogenous EcN PAMPs	Well-tolerated; no adverse effects	Induced IgA/IgG; blocked pseudovirus-ACE2 interaction	173	
*B. bronchiseptica*	*B. bronchiseptica* strain FX	WT-OMVs	Principal immunogenic Bb antigens	OMV-coated PEGylated nano-polysaccharide	Preclinical	BALB/c mice	SC: 5 µg OMV; 2 doses (D0, D14)	*R. glutinosa* polysaccharide (pRL)	No significant biochemical/weight changes	Potent Th1/Th2/Th17 response; reduced pulmonary colonization	174	
*P. aeruginosa*	*P. aeruginosa* clinical isolate PA14	WT-OMVs	OMV-surface proteins/lipids	OMV-coated STING nanocores	Preclinical	C57BL/6 mice	SC: 0.01-1 µg OMV; 3 doses (D0, D7, D14)	STING agonist (Mn/c-di-AMP)	Safe; no detectable organ damage	Fully protected against lethal pneumonia; robust IgG	175	
*S. aureus*	*E. coli*	WT-OMVs	OMV surface PAMPs	BMSC-OMV hybrid nanovesicles	Preclinical	Femoral osteomyelitis mice	IV: 50 µg/mL protein; 1 dose (D0)	Endogenous OMV PAMPs	Normal serum indicators; no organ pathology	Eradicated primary IAIs; prevented relapse; memory B cells	176	
MDR *E. coli* MRSA	Attenuated *E. coli*	b-OMVs	OmpA/OmpC/FijB; surface	OMV-coated PdIr nanozymes	Preclinical	MDR septic mice	IV: 50 µg/mL Pd; 1 dose (D0)	Endogenous OMV PAMPs	No systemic inflammation; biocompatible	Eradicated intracellular bacteria; 80% survival rate	177	
MRSA	*E. coli* MG1655* ∆msbB*	b-OMVs	Bacterial components	Siderophore-modified biomineralized OMV	Preclinical	KM/Balb/c mice	IV: Lsn 5 mg/kg + Mup 10 mg/kg; 3 doses (D0, D1, D2)	CaCO_3_ shell/Attenuated	Non-toxic to cells; no organ toxicity	Eradicated intracellular/extracellular bacteria; dual targeting	179	
*H. pylori*	Engineered *H. pylori* J99 (*ΔlpxEΔlpxFΔfutB* mutant)	b-OMVs	UreB, VacA, and CagA; surface	OMV/Hbp autotransporter platform	Preclinical	C57BL/6 mice	Oral: 2 doses; 4-week interval.	Self-adjuvanticity	Favorable safety profile; with no systemic or local toxicity in vivo and low in vitro cytotoxicity at relevant doses (>25 µg/mL).	*H. pylori* vaccine: potent mucosal immunity; Th17-biased response; reduced bacterial load & urease activity.	180	
Polymicrobial	*Bacillus Calmette–Guérin* (BCG)	WT-OMVs	Lipoproteins; surface	OMV	Preclinical	C57BL/6 mice	IP: 5 µg/g; single or multiple doses (3-day rest before challenge).	Self-adjuvanticity	Exceptional safety profile; characterized by LPS-free composition; 100% survival at high dose; and absence of chronic pathology.	OMV-trained immunity: superior to BCG; improved survival; reduced bacterial loads via enhanced myelopoiesis & macrophage function.	181	
SARS-CoV-2	Engineered *E. coli* BL21 (DE3) (S004) mutant	b-OMVs	RBD of SARS-CoV-2 S protein; surface	SpyTag/SpyCatcher (ST/SC) bioconjugation system	Preclinical	BALB/c mice	IN: 60 µg OMV + 1 µg RBD; 3 doses (D0, D14, D28); IM: same.	Self-adjuvanticity	Excellent safety profile; with normal serum biochemistry; no organ histopathology; and low hemolytic/cytotoxic activity.	OMV-RBD: high neutralizing Abs (>MF59); Th1-biased; IN route added mucosal immunity via DC activation (TLR3/NOD2).	182	
*C. jejuni* *S. TyphimuriumS. Enteritidis*	*C. jejuni* IDH2028*S. Typhimurium* PH94*S. Enteritidis* EVS111	WT-OMVs	LPS, immunogenic proteins, Outer-membrane proteins (OMPs); surface	OMV/TOMVs	Preclinical	BALB/c mice	IP: 5 µg; 3 doses, 14-day interval.	Self-adjuvanticity	Well tolerated in vivo; showing no significant organ damage or pathological changes.	TOMVs: long-term (180-day) protection vs. *C. jejuni*, *S. Typhimurium* and *S. Enteritidis*.	183	

b-OMVs: OMVs from bioengineered bacteria; BMSC: bone marrow mesenchymal stem cell; geGMMA: glycoengineered generalized modules for membrane antigens; GMMA: generalized modules for membrane antigens; HMVs: hybrid membrane vesicles; IM: intramuscular injection; IN: intranasal administration; IP: intraperitoneal injection; LOMVs: OMVs induced by exogenous Lysin P53; LPS: lipopolysaccharide; MDR: multidrug-resistant; MRSA: methicillin-resistant *Staphylococcus aureus*; nOMVs: naturally produced OMVs; OAg: O antigen; pRL: PEGylated nano-*Rehmannia glutinosa* polysaccharide; RBD: receptor-binding domain; SBA: serum bactericidal antibody; SC: subcutaneous injection; TOMVs: trivalent outer membrane vesicles; WT-OMVs: OMVs from wild-type bacteria.

## Abbreviations

AD: Alzheimer’s disease
AHR: aryl hydrocarbon receptor
APCs: antigen-presenting cells
BBB: blood–brain barrier
bEVs: bacterial extracellular vesicles
CaP: calcium phosphate
CFTR: cystic fibrosis transmembrane conductance regulator
ClyA: cytolysin A
CMVs: cytoplasmic membrane vesicles
CNP: cellular nanoparticle
CRC: colorectal cancer
cGAS: cyclic GMP-AMP synthase
CTLs: cytotoxic T lymphocytes
DAMPs: damage-associated molecular patterns
DCs: dendritic cells
EMVs: engineered OMVs
EMT: epithelial-mesenchymal transition
EOMVs: explosive outer membrane vesicles
GBM: glioblastoma
geGMMA: glycoengineered generalized modules for membrane antigens
GM-CSF: granulocyte-macrophage colony-stimulating factor
GMMA: generalized modules for membrane antigens
hBD-2: human β-defensin-2
HMVs: hybrid membrane vesicles
ICD: immunogenic cell death
ICG: indocyanine green
IDO: indoleamine 2,3-dioxygenase
IFN-γ: interferon-γ
LOMVs: lysin-induced OMVs
LPS: lipopolysaccharide
LTA: lipoteichoic acid
MDR: multidrug-resistant
MRSA: methicillin-resistant *Staphylococcus aureus*
OM: outer membrane
OIMVs: outer-inner membrane vesicles
OMVs: outer membrane vesicles
OSCC: oral squamous cell carcinoma
PADC: pancreatic ductal carcinoma
PAMPs: pathogen-associated molecular patterns
PBP2a: penicillin-binding protein 2a
PDAC: pancreatic ductal adenocarcinoma
PDA: polydopamine
PG: peptidoglycan
PLGA: poly(lactic-co-glycolic acid)
PmBF: polymyxin B-fluorescein
PQS: *Pseudomonas* quinolone signal
PTSLs: photosensitive thermosensitive liposomes
PTT: photothermal therapy
RA: rheumatoid arthritis
RBD: receptor-binding domain
ROS: reactive oxygen species
siRNA: small interfering RNA
SIRPα: signal regulatory protein-α
SPAb: domain B of staphylococcal protein A
SpyC: SpyCatcher
sRNAs: small RNAs
STING: stimulator of interferon genes
TAMs: tumor-associated macrophages
TDO: tryptophan-2,3-dioxygenase
TLR2: Toll-like receptor 2
TME: tumor microenvironment
TNBC: triple-negative breast cancer
TSMSs: tube-shaped membranous structures
αPD-L1: anti-programmed death-ligand 1 antibodies

## References

[1] Xie J, Li Q, Haesebrouck F, Van Hoecke L, Vandenbroucke RE (2022). The tremendous biomedical potential of bacterial extracellular vesicles. Trends Biotechnol.

[2] Toyofuku M, Nomura N, Eberl L (2019). Types and origins of bacterial membrane vesicles. Nat Rev Microbiol.

[3] Yu H, Lu Y, Lan F, Wang Y, Hu C, Mao L (2023). Engineering outer membrane vesicles to increase extracellular electron transfer of Shewanella oneidensis. ACS Synth Biol.

[4] Gerritzen MJH, Maas RHW, van den Ijssel J, van Keulen L, Martens DE, Wijffels RH (2018). High dissolved oxygen tension triggers outer membrane vesicle formation by Neisseria meningitidis. Microb Cell Fact.

[5] Roy R, You RI, Chang CH, Yang CY, Lin NT (2021). Carboxy-terminal processing protease controls production of outer membrane vesicles and biofilm in Acinetobacter baumannii. Microorganisms.

[6] Li Q, Li J, He T, Ji X, Wei R, Yu M (2024). Sub-MIC antibiotics modulate productions of outer membrane vesicles in tigecycline-resistant Escherichia coli. Antibiotics (Basel).

[7] Ojima Y, Sawabe T, Nakagawa M, Tahara YO, Miyata M, Azuma M (2021). Aberrant membrane structures in hypervesiculating Escherichia coli strain ΔmlaE ΔnlpI visualized by electron microscopy. Front Microbiol.

[8] Baeza N, Delgado L, Comas J, Mercade E (2021). Phage-mediated explosive cell lysis induces the formation of a different type of O-IMV in Shewanella vesiculosa M7(T). Front Microbiol.

[9] Chowdhury C, Jagannadham MV (2013). Virulence factors are released in association with outer membrane vesicles of Pseudomonas syringae pv. tomato T1 during normal growth. Biochim Biophys Acta.

[10] Tashiro Y, Inagaki A, Shimizu M, Ichikawa S, Takaya N, Nakajima-Kambe T (2011). Characterization of phospholipids in membrane vesicles derived from Pseudomonas aeruginosa. Biosci Biotechnol Biochem.

[11] Bonnington KE, Kuehn MJ (2016). Outer membrane vesicle production facilitates LPS remodeling and outer membrane maintenance in Salmonella during environmental transitions. mBio.

[12] Emerson LE, Mosby CA, Enslow S, Hui WW, Jones MK, Ferraro MJ (2024). Changes in lipid composition of host-derived extracellular vesicles following Salmonella infection. Microbiol Spectr.

[13] Rueter C, Bielaszewska M (2020). Secretion and delivery of intestinal pathogenic Escherichia coli virulence factors via outer membrane vesicles. Front Cell Infect Microbiol.

[14] Jeong GJ, Khan F, Tabassum N, Cho KJ, Kim YM (2024). Bacterial extracellular vesicles: modulation of biofilm and virulence properties. Acta Biomater.

[15] Saad MG, Beyenal H, Dong WJ (2024). Dual roles of the conditional extracellular vesicles derived from Pseudomonas aeruginosa biofilms: promoting and inhibiting bacterial biofilm growth. Biofilm.

[16] Dehinwal R, Cooley D, Rakov AV, Alugupalli AS, Harmon J, Cunrath O (2021). Increased production of outer membrane vesicles by Salmonella interferes with complement-mediated innate immune attack. mBio.

[17] Janda M, Rybak K, Krassini L, Meng C, Feitosa-Junior O, Stigliano E (2023). Biophysical and proteomic analyses of Pseudomonas syringae pv. tomato DC3000 extracellular vesicles suggest adaptive functions during plant infection. mBio.

[18] Deo P, Chow SH, Han ML, Speir M, Huang C, Schittenhelm RB (2020). Mitochondrial dysfunction caused by outer membrane vesicles from Gram-negative bacteria activates intrinsic apoptosis and inflammation. Nat Microbiol.

[19] David L, Taieb F, Pénary M, Bordignon PJ, Planès R, Bagayoko S (2022). Outer membrane vesicles produced by pathogenic strains of Escherichia coli block autophagic flux and exacerbate inflammasome activation. Autophagy.

[20] Bielaszewska M, Marejková M, Bauwens A, Kunsmann-Prokscha L, Mellmann A, Karch H (2018). Enterohemorrhagic Escherichia coli O157 outer membrane vesicles induce interleukin 8 production in human intestinal epithelial cells by signaling via Toll-like receptors TLR4 and TLR5 and activation of the nuclear factor NF-κB. Int J Med Microbiol.

[21] Trampert DC (2025). Gut bacterial membrane components as pathogenic signalling molecules in PSC-IBD. J Hepatol.

[22] Tan J, Ni D, Taitz J, Pinget GV, Read M, Senior A (2022). Dietary protein increases T-cell-independent sIgA production through changes in gut microbiota-derived extracellular vesicles. Nat Commun.

[23] Yang Y, Yang L, Yang Y, Deng H, Su S, Xia Y (2025). Bacteroides fragilis-derived outer membrane vesicles deliver miR-5119 and alleviate colitis by targeting PD-L1 to inhibit GSDMD-mediated neutrophil extracellular trap formation. Adv Sci (Weinh).

[24] Older EA, Mitchell MK, Campbell A, Lian X, Madden M, Wang Y (2025). Human gut commensal Alistipes timonensis modulates the host lipidome and delivers anti-inflammatory outer membrane vesicles to suppress colitis in an Il10-deficient mouse model. Gut Microbes.

[25] Brown EM, Temple ER, Jeanfavre S, Avila-Pacheco J, Taylor N, Liu K (2025). Bacteroides sphingolipids promote anti-inflammatory responses through the mevalonate pathway. Cell Host Microbe.

[26] Yang T, Hu X, Cao F, Yun F, Jia K, Zhang M (2025). Targeting symbionts by apolipoprotein L proteins modulates gut immunity. Nature.

[27] Marchant P, Carreño A, Vivanco E, Silva A, Nevermann J, Otero C (2021). "One for all": functional transfer of OMV-mediated polymyxin B resistance from Salmonella enterica sv. Typhi ΔtolR and ΔdegS to susceptible bacteria. Front Microbiol.

[28] Zingl FG, Kohl P, Cakar F, Leitner DR, Mitterer F, Bonnington KE (2020). Outer membrane vesiculation facilitates surface exchange and in vivo adaptation of Vibrio cholerae. Cell Host Microbe.

[29] Zingl FG, Thapa HB, Scharf M, Kohl P, Müller AM, Schild S (2021). Outer membrane vesicles of Vibrio cholerae protect and deliver active cholera toxin to host cells via porin-dependent uptake. mBio.

[30] Engevik MA, Danhof HA, Ruan W, Engevik AC, Chang-Graham AL, Engevik KA (2021). Fusobacterium nucleatum secretes outer membrane vesicles and promotes intestinal inflammation. mBio.

[31] Baryalai P, Irenaeus D, Toh E, Ramstedt M, Uhlin BE, Nadeem A (2025). Hemagglutinin protease HapA associated with Vibrio cholerae outer membrane vesicles (OMVs) disrupts tight and adherens junctions. J Extracell Vesicles.

[32] Xing J, Niu T, Yu T, Zou B, Shi C, Wang Y (2025). Faecalibacterium prausnitzii-derived outer membrane vesicles reprogram gut microbiota metabolism to alleviate porcine epidemic diarrhea virus infection. Microbiome.

[33] Zheng X, Gong T, Luo W, Hu B, Gao J, Li Y (2024). Fusobacterium nucleatum extracellular vesicles are enriched in colorectal cancer and facilitate bacterial adhesion. Sci Adv.

[34] Chmiela M, Walczak N, Rudnicka K (2018). Helicobacter pylori outer membrane vesicles involvement in the infection development and Helicobacter pylori-related diseases. J Biomed Sci.

[35] Jin S, Lu Y, Zuo Y, Xu Q, Hao Y, Zuo H (2025). Akkermansia muciniphila ameliorates chronic stress-induced colorectal tumor growth by releasing outer membrane vesicles. Gut Microbes.

[36] Nonaka S, Kadowaki T, Nakanishi H (2022). Secreted gingipains from Porphyromonas gingivalis increase permeability in human cerebral microvascular endothelial cells through intracellular degradation of tight junction proteins. Neurochem Int.

[37] Chuang WC, Yang CN, Wang HW, Lin SK, Yu CC, Syu JH (2024). The mechanisms of Porphyromonas gingivalis-derived outer membrane vesicles-induced neurotoxicity and microglia activation. J Dent Sci.

[38] Singhrao SK, Olsen I (2018). Are Porphyromonas gingivalis outer membrane vesicles microbullets for sporadic Alzheimer's disease manifestation?. J Alzheimers Dis Rep.

[39] Wei S, Peng W, Mai Y, Li K, Wei W, Hu L (2020). Outer membrane vesicles enhance tau phosphorylation and contribute to cognitive impairment. J Cell Physiol.

[40] Han EC, Choi SY, Lee Y, Park JW, Hong SH, Lee HJ (2019). Extracellular RNAs in periodontopathogenic outer membrane vesicles promote TNF-α production in human macrophages and cross the blood-brain barrier in mice. FASEB J.

[41] Cao Y, Li X, Jing L, Li F, Chen Y, Deng D (2026). Targeted inhibition of P. gingivalis OMV-derived TsRNA by tetrahedral framework nucleic acids promotes periodontal regeneration. J Nanobiotechnology.

[42] Park AM, Tsunoda I (2022). Helicobacter pylori infection in the stomach induces neuroinflammation: the potential roles of bacterial outer membrane vesicles in an animal model of Alzheimer's disease. Inflamm Regen.

[43] Chen C, Zhu X, Song Z, Xing Y, Jin W, Li F (2025). Dual-targeted bacterial outer membrane vesicles enhance glioblastoma immunotherapy by regulating tumor microenvironment and inducing IFN-γ-mediated ferroptosis. J Nanobiotechnology.

[44] Pan J, Wang Z, Huang X, Xue J, Zhang S, Guo X (2023). Bacteria-derived outer-membrane vesicles hitchhike neutrophils to enhance ischemic stroke therapy. Adv Mater.

[45] Li P, Peng T, Xiang T, Luo W, Liao W, Wei DD (2023). Klebsiella pneumoniae outer membrane vesicles induce strong IL-8 expression via NF-κB activation in normal pulmonary bronchial cells. Int Immunopharmacol.

[46] Dell'Annunziata F, Ciaglia E, Folliero V, Lopardo V, Maciag A, Galdiero M (2024). Klebsiella pneumoniae-OMVs activate death-signaling pathways in human bronchial epithelial host cells (BEAS-2B). Heliyon.

[47] Xie Y, Shi YH, Wang LL, Li CW, Wu M, Xu JF (2025). Outer membrane vesicle contributes to the Pseudomonas aeruginosa resistance to antimicrobial peptides in the acidic airway of bronchiectasis patients. MedComm (2020).

[48] Jung AL, Stoiber C, Herkt CE, Schulz C, Bertrams W, Schmeck B (2016). Legionella pneumophila-derived outer membrane vesicles promote bacterial replication in macrophages. PLoS Pathog.

[49] Yao Q, Liu T, Wen J, Yang Q, Li Y, Yan H (2025). SpyTag-PEGylated probiotics delivering IL-1Ra modulate gut-lung crosstalk to mitigate septic lung injury. J Control Release.

[50] Zhu W, Han L, He L, Wei S, Li J, Xin L (2026). Parabacteroides goldsteinii-derived outer membrane vesicles alleviate acute lung injury via modulation of bile acid metabolism. J Nanobiotechnology.

[51] Yang D, Chen X, Wang J, Lou Q, Lou Y, Li L (2019). Dysregulated lung commensal bacteria drive interleukin-17B production to promote pulmonary fibrosis through their outer membrane vesicles. Immunity.

[52] Bomberger JM, Ye S, Maceachran DP, Koeppen K, Barnaby RL, O'Toole GA (2011). A Pseudomonas aeruginosa toxin that hijacks the host ubiquitin proteolytic system. PLoS Pathog.

[53] Tan TT, Morgelin M, Forsgren A, Riesbeck K (2007). Haemophilus influenzae survival during complement-mediated attacks is promoted by Moraxella catarrhalis outer membrane vesicles. J Infect Dis.

[54] Augustyniak D, Roszkowiak J, Wiśniewska I, Skała J, Gorczyca D, Drulis-Kawa Z (2018). Neuropeptides SP and CGRP diminish the Moraxella catarrhalis outer membrane vesicle- (OMV-) triggered inflammatory response of human A549 epithelial cells and neutrophils. Mediators Inflamm.

[55] Lin Y, Wang J, Bu F, Zhang R, Wang J, Wang Y (2025). Bacterial extracellular vesicles in the initiation, progression and treatment of atherosclerosis. Gut Microbes.

[56] Yin S, Qiao Q, Li Z, Lu L, Jiang M, Gao H (2026). Porphyromonas gingivalis-derived outer membrane vesicles promote vascular endothelial glycocalyx injury via the PPAD/CitH3/B3GAT1 pathway. J Nanobiotechnology.

[57] Yang S, Xia YP, Luo XY, Chen SL, Li BW, Ye ZM (2019). Exosomal CagA derived from Helicobacter pylori-infected gastric epithelial cells induces macrophage foam cell formation and promotes atherosclerosis. J Mol Cell Cardiol.

[58] Vanaja SK, Russo AJ, Behl B, Banerjee I, Yankova M, Deshmukh SD (2016). Bacterial outer membrane vesicles mediate cytosolic localization of LPS and caspase-11 activation. Cell.

[59] Chen G, Gao C, Jiang S, Cai Q, Li R, Sun Q (2024). Fusobacterium nucleatum outer membrane vesicles activate autophagy to promote oral cancer metastasis. J Adv Res.

[60] Kew C, Prieto-Garcia C, Bhattacharya A, Tietgen M, MacNair CR, Carfrae LA (2024). The aryl hydrocarbon receptor and FOS mediate cytotoxicity induced by Acinetobacter baumannii. Nat Commun.

[61] Toh E, Baryalai P, Nadeem A, Aung KM, Myint SL, Zlatkov N (2025). Sublytic activity of a pore-forming protein from commensal bacteria causes epigenetic modulation of tumour-affiliated protein expression. J Extracell Vesicles.

[62] Shangguan W, Li W, Huang W, Wu J, Yu Y, Huang Y (2026). FomA-containing outer membrane vesicles of Fusobacterium nucleatum facilitate bladder cancer lymphatic metastasis via IL-6-dependent M2b macrophage polarization. Adv Sci (Weinh).

[63] Liao X, Si H, Lai Y, Zhang X, Feng Y, Zhou T (2025). Porphyromonas gingivalis-OMVs promote the epithelial-mesenchymal transition of oral squamous cell carcinoma by inhibiting ferroptosis through the NF-κB pathway. J Oral Microbiol.

[64] Liu D, Liu S, Liu J, Miao L, Zhang S, Pan Y (2021). sRNA23392 packaged by Porphyromonas gingivalis outer membrane vesicles promotes oral squamous cell carcinomas migration and invasion by targeting desmocollin-2. Mol Oral Microbiol.

[65] Lamprinaki D, Garcia-Vello P, Marchetti R, Hellmich C, McCord KA, Bowles KM (2021). Siglec-7 mediates immunomodulation by colorectal cancer-associated Fusobacterium nucleatum ssp. animalis. Front Immunol.

[66] Li W, Zhang Z, Wu R, Mao M, Ji Y, Wang X (2025). Fusobacterium nucleatum-derived outer membrane vesicles promote immunotherapy resistance via changes in tryptophan metabolism in tumour-associated macrophages. J Extracell Vesicles.

[67] Xiao Z, Yuan J, Wu Q, Qin J, Liu Y, Zhang S (2026). Engineered bacterial extracellular vesicles mediate pyroptosis to counteract m(6)A methylation-based immunosuppression after insufficient radiofrequency ablation of hepatocellular carcinoma. Acta Pharm Sin B.

[68] Meng R, Zeng M, Ji Y, Huang X, Xu M (2023). The potential role of gut microbiota outer membrane vesicles in colorectal cancer. Front Microbiol.

[69] Liang A, Korani L, Yeung CLS, Tey SK, Yam JWP (2024). The emerging role of bacterial extracellular vesicles in human cancers. J Extracell Vesicles.

[70] Mekata M, Yoshida K, Takai A, Hiroshima Y, Ikuta A, Seyama M (2025). Porphyromonas gingivalis outer membrane vesicles increase vascular permeability by inducing stress fiber formation and degrading vascular endothelial-cadherin in endothelial cells. FEBS J.

[71] Seyama M, Yoshida K, Yoshida K, Fujiwara N, Ono K, Eguchi T (2020). Outer membrane vesicles of Porphyromonas gingivalis attenuate insulin sensitivity by delivering gingipains to the liver. Biochim Biophys Acta Mol Basis Dis.

[72] Su D, Li M, Xie Y, Xu Z, Lv G, Jiu Y (2025). Gut commensal bacteria Parabacteroides goldsteinii-derived outer membrane vesicles suppress skin inflammation in psoriasis. J Control Release.

[73] Li L, Li XQ, Zhang JX, Wu QY, Chen PP, Ruan XZ (2026). Single-chain anti-IL-1β antibody carried by outer membrane vesicles of Bacteroides fragilis alleviates tubular inflammation in chronic kidney disease. J Extracell Vesicles.

[74] Kim HY, Song MK, Gho YS, Kim HH, Choi BK (2021). Extracellular vesicles derived from the periodontal pathogen Filifactor alocis induce systemic bone loss through Toll-like receptor 2. J Extracell Vesicles.

[75] Kim HY, Song MK, Lim Y, Jang JS, An SJ, Kim HH (2022). Effects of extracellular vesicles derived from oral bacteria on osteoclast differentiation and activation. Sci Rep.

[76] Luo X, Wang Y, Ning T, Lei Q, Cui H, Zou X (2025). Outer membrane vesicles of Porphyromonas gingivalis impede bone regeneration by inducing ferroptosis via the Hippo-YAP signaling pathway. J Nanobiotechnology.

[77] Cai D, Li Z, Gao W, Liu J, Qi X, Jin J (2025). Proinflammatory macrophage-targeted nanoparticles rejuvenate aged macrophages and their phagocytic capacity. ACS Nano.

[78] Hong M, Li Z, Liu H, Zheng S, Zhang F, Zhu J (2023). Fusobacterium nucleatum aggravates rheumatoid arthritis through FadA-containing outer membrane vesicles. Cell Host Microbe.

[79] Coats SR, Su TH, Luderman Miller Z, King AJ, Ortiz J, Reddy A (2025). Porphyromonas gingivalis outer membrane vesicles divert host innate immunity and promote inflammation via C4' monophosphorylated lipid A. J Immunol.

[80] Wu N, Han Z, Lv W, Huang Y, Zhu J, Deng J (2025). Reprogramming peritoneal macrophages with outer membrane vesicle-coated PLGA nanoparticles for endometriosis prevention. Biomaterials.

[81] Folliero V, Santonastaso M, Dell'Annunziata F, De Franciscis P, Boccia G, Colacurci N (2022). Impact of Escherichia coli outer membrane vesicles on sperm function. Pathogens.

[82] Gao H, Gao Y, Yang C, Dong D, Yang J, Peng G (2018). Influence of outer membrane vesicles of Proteus mirabilis isolated from boar semen on sperm function. Vet Microbiol.

[83] Wei Y, Zhou L, Zhao X, Qiu H, Hu D, Shi Z (2025). Helicobacter pylori outer membrane vesicles mediate central tolerance in C57BL/6J mice offspring T cells via maternal-fetal transmission. Front Immunol.

[84] Lara B, Loureiro I, Gliosca L, Castagnola L, Merech F, Gallino L (2023). Porphyromonas gingivalis outer membrane vesicles shape trophoblast cell metabolism impairing functions associated to adverse pregnancy outcome. J Cell Physiol.

[85] Bradley AJ, Mashburn-Warren L, Blalock LC, Scarpetti F, Lauber CL (2025). Porphyromonas gingivalis outer membrane vesicles alter cortical neurons and Tau phosphorylation in the embryonic mouse brain. PLoS One.

[86] Garling A, Auvray F, Epardaud M, Oswald É, Branchu P (2025). Outer membrane vesicles as a versatile platform for vaccine development: engineering strategies, applications and challenges. J Extracell Vesicles.

[87] Gerritzen MJH, Martens DE, Wijffels RH, van der Pol L, Stork M (2017). Bioengineering bacterial outer membrane vesicles as vaccine platform. Biotechnol Adv.

[88] Calvaresi V, Dello Iacono L, Borghi S, Luzzi E, Biolchi A, Ferlenghi I (2026). Structural dynamics and immunogenicity of the recombinant and outer membrane vesicle-embedded meningococcal antigen NadA. Nat Commun.

[89] Zheng K, Feng Y, Li L, Kong F, Gao J, Kong X (2024). Engineered bacterial outer membrane vesicles: a versatile bacteria-based weapon against gastrointestinal tumors. Theranostics.

[90] Bai X, Li C, Qiu J, Wu L, Liu X, Yin T (2025). A "plug-and-display" nanoparticle based on attenuated outer membrane vesicles enhances the immunogenicity of protein antigens. J Control Release.

[91] Wang D, Li B, Zhang D, Zhuang J, Chen S, Tian J (2025). Amplifying engineered bacterial outer membrane vesicle production using functional peptidoglycan inhibitors. J Am Chem Soc.

[92] Liu K, Du S, Yang J, Li J, Wang S, Zhang Z (2024). Engineered bacterial membrane vesicle as safe and efficient nano-heaters to reprogram tumor microenvironment for enhanced immunotherapy. J Control Release.

[93] Nie W, Jiang A, Ou X, Zhou J, Li Z, Liang C (2024). Metal-polyphenol "prison" attenuated bacterial outer membrane vesicle for chemodynamics promoted in situ tumor vaccines. Biomaterials.

[94] Ginez LD, Osorio A, Correal-Medina V, Arenas T, González-Espinosa C, Camarena L (2025). Outer membrane vesicles from Caulobacter crescentus: a platform for recombinant antigen presentation. ACS Nano.

[95] Zanella I, König E, Tomasi M, Gagliardi A, Frattini L, Fantappiè L (2021). Proteome-minimized outer membrane vesicles from Escherichia coli as a generalized vaccine platform. J Extracell Vesicles.

[96] Li C, Xue H, Du X, Nyaruaba R, Yang H, Wei H (2024). Outer membrane vesicles generated by an exogenous bacteriophage lysin and protection against Acinetobacter baumannii infection. J Nanobiotechnology.

[97] Nie X, Shi C, Chen X, Yu C, Jiang Z, Xu G (2023). A single-shot prophylactic tumor vaccine enabled by an injectable biomembrane hydrogel. Acta Biomater.

[98] Zhao X, Zhao R, Nie G (2022). Nanocarriers based on bacterial membrane materials for cancer vaccine delivery. Nat Protoc.

[99] Luo S, Yang Y, Chen L, Kannan PR, Yang W, Zhang Y (2024). Outer membrane vesicle-wrapped manganese nanoreactor for augmenting cancer metalloimmunotherapy through hypoxia attenuation and immune stimulation. Acta Biomater.

[100] Zeng J, Fang X, Li Y, Yan Q, Li Y, Yu H (2026). Remodeling the tumor microenvironment: an emerging paradigm for reinvigorating immunologically cold tumors and advancing cancer immunotherapy. ACS Nano.

[101] Tang M, Lv W, Gao X, Xu C, Zhang T, Liang B (2026). Immunological conversion of solid tumors using an outer membrane vesicle based checkpoint nanoinhibitor for cancer immunotherapy. ACS Nano.

[102] Guo Q, Li X, Zhou W, Chu Y, Chen Q, Zhang Y (2021). Sequentially triggered bacterial outer membrane vesicles for macrophage metabolism modulation and tumor metastasis suppression. ACS Nano.

[103] Ban W, Sun M, Huang H, Huang W, Pan S, Liu P (2023). Engineered bacterial outer membrane vesicles encapsulating oncolytic adenoviruses enhance the efficacy of cancer virotherapy by augmenting tumor cell autophagy. Nat Commun.

[104] Qing S, Lyu C, Zhu L, Pan C, Wang S, Li F (2020). Biomineralized bacterial outer membrane vesicles potentiate safe and efficient tumor microenvironment reprogramming for anticancer therapy. Adv Mater.

[105] Lin YH, Chen CW, Chen MY, Xu L, Tian X, Cheung SH (2025). The bacterial outer membrane vesicle-cloaked immunostimulatory nanoplatform reinvigorates T cell function and reprograms tumor immunity. ACS Nano.

[106] Chen Z, Wang B, Zheng J, Liu C, Xu P, Zhou Q (2025). Reprogramming tumor-associated macrophages and blocking PD-L1 via engineered outer membrane vesicles to enhance T cell infiltration and cytotoxic functions. J Nanobiotechnology.

[107] Li QR, Zhang X, Zhang C, Zhang Y, Niu MT, Chen Z (2025). Biomineralized engineered bacterial outer membrane vesicles as cGAS-STING nanoagonists synergize with lactate metabolism modulation to potentiate immunotherapy. J Am Chem Soc.

[108] Peng F, Lei Z, Zhang Z, Su Z, Zhang C, Yang H (2026). Engineered bacteria-vesicle delivered lactate reprogramming boosts tumor radiosensitivity. Adv Sci (Weinh).

[109] Xia P, Qu C, Xu X, Tian M, Li Z, Ma J (2025). Nanobody engineered and photosensitiser loaded bacterial outer membrane vesicles potentiate antitumour immunity and immunotherapy. J Extracell Vesicles.

[110] Zhang F, Li Q, Dai H, Li W, Chen X, Wu H (2025). Chimeric nanozyme bacterial outer membrane vesicles reprograming tumor microenvironment for safe and efficient anticancer therapy. Adv Sci (Weinh).

[111] Liu J, Zhao H, Gao T, Huang X, Liu S, Liu M (2024). Glypican-3-targeted macrophages delivering drug-loaded exosomes offer efficient cytotherapy in mouse models of solid tumours. Nat Commun.

[112] Wang Y, Fan Y, Zhang X, Liu J, Sun D, Li L (2025). In situ production and precise release of bioactive GM-CSF and siRNA by engineered bacteria for macrophage reprogramming in cancer immunotherapy. Biomaterials.

[113] An JX, Qin YT, Tang Y, He JL, Zhang C, Rao ZY (2025). Polymerization of L-arginine into nanomicelles for immunometabolic engineering of adoptive macrophages in solid tumor therapy. Angew Chem Int Ed Engl.

[114] Wang D, Liu C, You S, Zhang K, Li M, Cao Y (2020). Bacterial vesicle-cancer cell hybrid membrane-coated nanoparticles for tumor specific immune activation and photothermal therapy. ACS Appl Mater Interfaces.

[115] Zhai Y, Ma Y, Pang B, Zhang J, Li Y, Rui Y (2021). A cascade targeting strategy based on modified bacterial vesicles for enhancing cancer immunotherapy. J Nanobiotechnology.

[116] Zhao C, Pan Y, Cao L, Tang Y, Chen X, Zhao X (2025). Metal-phenolic outer membrane vesicles for cancer radioimmunotherapy. J Am Chem Soc.

[117] Luo Y, Wang Y, Liu B, Liu Y, Zhang W, Chen S (2025). A "CPApoptosis" nano-actuator switches immune-off solid tumors to immune-on for fueling T-cell-based immunotherapy. Theranostics.

[118] Lötvall J, Ordouzadeh N, Crescitelli R, Deshmukh M, Jin T, Park KS (2025). STING agonist drug delivery by bacterial extracellular vesicles induces synergistic immuno-oncology responses and efficient inhibition of tumour growth. J Extracell Vesicles.

[119] Guan M, Xie XT, Zhou D, Cheng K, Zhang B, Xu XY (2025). Engineered bacterial outer membrane vesicles hitchhiking on neutrophils for antibody drug delivery to enhance postoperative immune checkpoint therapy. Adv Sci (Weinh).

[120] Sun JX, Ma SY, Xu JY, Abudureyimu M, An Y, Xu JZ (2025). Escherichia coli Nissle 1917 outer membrane vesicles encapsulating oncolytic virus remodel tumor-associated macrophages and kill prostate cancer cells. J Control Release.

[121] Wang H, Zhan H, Pan B, Zeng L, Chen Z, Liu S (2025). Engineering CRISPR system-based bacterial outer membrane vesicle potentiates T cell immunity for enhanced cancer immunotherapy. Adv Mater.

[122] Chen Q, Huang G, Wu W, Wang J, Hu J, Mao J (2020). A hybrid eukaryotic-prokaryotic nanoplatform with photothermal modality for enhanced antitumor vaccination. Adv Mater.

[123] Wang M, Yan G, Xiao Q, Zhou N, Chen HR, Xia W (2023). Iontophoresis-driven microneedle arrays delivering transgenic outer membrane vesicles in program that stimulates transcutaneous vaccination for cancer immunotherapy. Small Sci.

[124] Pan J, Li X, Shao B, Xu F, Huang X, Guo X (2022). Self-blockade of PD-L1 with bacteria-derived outer-membrane vesicle for enhanced cancer immunotherapy. Adv Mater.

[125] Cui C, He Q, Wang J, Kang J, Ma W, Nian Y (2023). Targeted miR-34a delivery with PD1 displayed bacterial outer membrane vesicles-coated zeolitic imidazolate framework nanoparticles for enhanced tumor therapy. Int J Biol Macromol.

[126] Zhang R, Zhang B, Duan S, Xu X, Jiang R, Zhao Y (2026). Biosynthetic OMVs with endogenous GM-CSF loading for ultrasound-triggered in situ cancer vaccination. J Nanobiotechnology.

[127] Xu W, Wei L, Zhao X, Zhao Y, Zhang T, Zhang J (2026). Pulmonary delivery of glycine-induced outer membrane vesicles as in situ vaccines for metastatic lung cancer. J Control Release.

[128] Han D, Wang F, Ma Y, Zhao Y, Zhang W, Zhang Z (2023). Redirecting antigens by engineered photosynthetic bacteria and derived outer membrane vesicles for enhanced cancer immunotherapy. ACS Nano.

[129] Yu C, Hu Z, Hu G, Jia Q, Xiao Y, Ahmad H (2026). NIR-II fluorescence membrane probes for rapidly labelling hybrid of probiotic outer membrane vesicles and anti-PD-L1 scFv over-expressing cellular vesicles with targeted photothermal-immunotherapy of colon cancer. Biomaterials.

[130] Liu X, Niu Q, Yang H, Du T, Liu Y, Zhou H (2025). Outer membrane vesicles-based nano-regulator for cancer immunotherapy by modulating neutrophils N1 polarization and hitchhiking delivery. J Nanobiotechnology.

[131] Cheng L, Peng D, Liu Z, Tang J, Zhang P, Li M (2026). Engineered bacterial outer membrane vesicles enhanced tumor immunotherapy through remodeling tumor stroma and targeted delivery of CD73 siRNA. Biomaterials.

[132] Li Y, Cong Z, Xie L, Tang S, Ren C, Peng X (2023). Magnetically powered immunogenic macrophage microrobots for targeted multimodal cancer therapy. Small.

[133] Zhou J, Karshalev E, Mundaca-Uribe R, Esteban-Fernández de Ávila B, Krishnan N, Xiao C (2021). Physical disruption of solid tumors by immunostimulatory microrobots enhances antitumor immunity. Adv Mater.

[134] You H, Zhang S, Zhang Y, Chen Q, Wu Y, Zhou Z (2025). Engineered bacterial outer membrane vesicles-based doxorubicin and CD47-siRNA co-delivery nanoplatform overcomes immune resistance to potentiate the immunotherapy of glioblastoma. Adv Mater.

[135] Sun D, Li Y, Yin X, Fan Y, Liu J, Wang Y (2024). Utilizing engineered bacteria as "cell factories" in vivo for intracellular RNA-loaded outer membrane vesicles' self-assembly in tumor treatment. ACS Nano.

[136] Li X, Guo Q, Chen Q, Chu Y, Zhang Y, Chen H (2024). Reconciling the cooperative-competitive patterns among tumor and immune cells for triple-negative breast cancer treatment using multimodule nanocomplexes. Adv Mater.

[137] Qin J, Liu J, Wei Z, Li X, Chen Z, Li J (2025). Targeted intervention in nerve-cancer crosstalk enhances pancreatic cancer chemotherapy. Nat Nanotechnol.

[138] Ji P, Wu P, Wang L, Wang Y, Guo X, Gao R (2024). Lysosome-targeting bacterial outer membrane vesicles for tumor specific degradation of PD-L1. Small.

[139] Zhang J, Wan S, Zhou H, Du J, Li Y, Zhu H (2024). Programmed nanocloak of commensal bacteria-derived nanovesicles amplify strong immunoreactivity against tumor growth and metastatic progression. ACS Nano.

[140] Tong Q, Li K, Huang F, Dai Y, Zhang T, Muaibati M (2023). Extracellular vesicles hybrid plasmid-loaded lipid nanovesicles for synergistic cancer immunotherapy. Mater Today Bio.

[141] Zhuang WR, Wang Y, Nie W, Lei Y, Liang C, He J (2023). Bacterial outer membrane vesicle based versatile nanosystem boosts the efferocytosis blockade triggered tumor-specific immunity. Nat Commun.

[142] Wang W, Xu H, Ye Q, Tao F, Wheeldon I, Yuan A (2022). Systemic immune responses to irradiated tumours via the transport of antigens to the tumour periphery by injected flagellate bacteria. Nat Biomed Eng.

[143] Wang S, Chen CC, Hu MH, Cheng M, Tu HF, Tsai YC (2024). Arginine-linked HPV-associated E7 displaying bacteria-derived outer membrane vesicles as a potent antigen-specific cancer vaccine. J Transl Med.

[144] Liang J, Cheng K, Li Y, Xu J, Chen Y, Ma N (2022). Personalized cancer vaccines from bacteria-derived outer membrane vesicles with antibody-mediated persistent uptake by dendritic cells. Fundam Res.

[145] Cheng K, Zhao R, Li Y, Qi Y, Wang Y, Zhang Y (2021). Bioengineered bacteria-derived outer membrane vesicles as a versatile antigen display platform for tumor vaccination via Plug-and-Display technology. Nat Commun.

[146] Park KS, Svennerholm K, Crescitelli R, Lässer C, Gribonika I, Lötvall J (2021). Synthetic bacterial vesicles combined with tumour extracellular vesicles as cancer immunotherapy. J Extracell Vesicles.

[147] Lu Y, Ma N, Cheng K, Liu G, Liang J, Xu C (2025). An OMV-based nanovaccine as antigen presentation signal enhancer for cancer immunotherapy. Adv Mater.

[148] Liang J, Zhu F, Cheng K, Ma N, Ma X, Feng Q (2023). Outer membrane vesicle-based nanohybrids target tumor-associated macrophages to enhance trained immunity-related vaccine-generated antitumor activity. Adv Mater.

[149] Qin H, Li H, Zhu J, Qin Y, Li N, Shi J (2023). Biogenetic vesicle-based cancer vaccines with tunable surface potential and immune potency. Small.

[150] Li Y, Ma X, Yue Y, Zhang K, Cheng K, Feng Q (2022). Rapid surface display of mRNA antigens by bacteria-derived outer membrane vesicles for a personalized tumor vaccine. Adv Mater.

[151] Li Y, Zhang K, Wu Y, Yue Y, Cheng K, Feng Q (2022). Antigen capture and immune modulation by bacterial outer membrane vesicles as in situ vaccine for cancer immunotherapy post-photothermal therapy. Small.

[152] Chen L, Ma S, Wu H, Zheng L, Yi Y, Liu G (2025). Zonated copper-driven breast cancer progression countered by a copper-depleting nanoagent for immune and metabolic reprogramming. Adv Sci (Weinh).

[153] Xerri NL, Payne SM (2022). Bacteroides thetaiotaomicron outer membrane vesicles modulate virulence of Shigella flexneri. mBio.

[154] Burt M, Angelidou G, Mais CN, Preußer C, Glatter T, Heimerl T (2024). Lipid A in outer membrane vesicles shields bacteria from polymyxins. J Extracell Vesicles.

[155] Zhu X, Zou A, Zhao M, Hou J, Xianyu Y (2025). Probiotic vesicles-implemented multifunctional nanotherapeutic approach for antibacterial, anti-inflammatory, and tissue regeneration in bacterial-infected oral ulcer healing. Adv Healthc Mater.

[156] Gurunathan S, Thangaraj P, Das J, Kim JH (2023). Antibacterial and antibiofilm effects of Pseudomonas aeruginosa derived outer membrane vesicles against Streptococcus mutans. Heliyon.

[157] Miri S, Mottawea W, Leao L, Chiba M, Li Y, Minic Z (2025). Ligilactobacillus-derived extracellular vesicles inhibit growth and virulence of enteric pathogens. Probiotics Antimicrob Proteins.

[158] Pourtalebi Jahromi L, Kronast B, Munkert J, Sana L, Koch M, Danzer H (2026). Bioengineered bacterial vesicles and biomimetic hybrids eliminate biofilms and balance the gut microbiome. Small.

[159] Zhang X, Qian C, Tang M, Zeng W, Kong J, Fu C (2023). Carbapenemase-loaded outer membrane vesicles protect Pseudomonas aeruginosa by degrading imipenem and promoting mutation of antimicrobial resistance gene. Drug Resist Updat.

[160] Monteiro R, Alcantud BS, Piersma S, Hendrickx APA, Maaß S, Becher D (2025). Outer membrane vesicles of carbapenem-resistant clinical Acinetobacter baumannii isolates protect both the vesicle-producing bacteria and non-resistant bacteria against carbapenems. Microbiol Res.

[161] Zhao M, He M, Lin X, Wu K, Yang F, Chen X (2025). Identification of outer membrane vesicles as a new vehicle mediating antibiotic resistance gene transfer in Campylobacter. J Extracell Vesicles.

[162] Chen Z, Liu Y, Jiang L, Zhang C, Qian X, Gu J (2024). Bacterial outer membrane vesicles increase polymyxin resistance in Pseudomonas aeruginosa while inhibiting its quorum sensing. J Hazard Mater.

[163] Baker S, Krishna A, Higham S, Naydenova P, O'Leary S, Scott JB (2024). Exploiting human immune repertoire transgenic mice for protective monoclonal antibodies against antimicrobial resistant Acinetobacter baumannii. Nat Commun.

[164] Zavan L, Hor L, Johnston EL, Paxman J, Heras B, Kaparakis-Liaskos M (2025). Antigen 43 associated with Escherichia coli membrane vesicles contributes to bacterial cell association and biofilm formation. Microbiol Spectr.

[165] Yang Y, Zeng Y, Zhu J, Li J, Gu L, Wei L (2024). OMP38 of carbapenem-resistant Acinetobacter baumannii-mediated mtDNA release activates the cGAS-STING signaling to induce inflammatory response. Adv Sci (Weinh).

[166] Semchenko EA, Seib KL (2022). Outer membrane vesicle vaccines for Neisseria gonorrhoeae. Nat Rev Urol.

[167] Hanumunthadu B, Demissie T, Greenland M, Skidmore P, Tanha K, Crocker-Buque T (2025). Safety and immunogenicity of the invasive non-typhoidal Salmonella (iNTS)-GMMA vaccine: a first-in-human, randomised, dose escalation trial. EBioMedicine.

[168] Higham SL, Baker S, Flight KE, Krishna A, Kellam P, Reece ST (2023). Intranasal immunization with outer membrane vesicles (OMV) protects against airway colonization and systemic infection with Acinetobacter baumannii. J Infect.

[169] Ye J, Huang W, Yu S, Guo Y, Sun P, Chen Z (2025). Development of a promising bivalent vaccine against Klebsiella pneumoniae based on glycoengineered GMMA (geGMMA). Exploration (Beijing).

[170] Peng X, Luo Y, Yang L, Yang YY, Yuan P, Chen X (2024). A multiantigenic antibacterial nanovaccine utilizing hybrid membrane vesicles for combating Pseudomonas aeruginosa infections. J Extracell Vesicles.

[171] Huang Y, Wang H, Peng X, Li T, Fan X, Shen Q (2025). Biological nanoparticles from Brucella abortus (ΔeipB∆perΔwadC) elicit protective immunity against brucellosis. J Nanobiotechnology.

[172] Yang Y, Xu L, Li Y, Sun Y, Tang Y, Xiao Z (2025). High-yield outer membrane vesicles derived from probiotics as a nanoplatform for precise treatment and prophylaxis of Pseudomonas aeruginosa infection. J Extracell Vesicles.

[173] Kamble NS, Thomas S, Madaan T, Ehsani N, Sange S, Tucker K (2025). Engineered bacteria as an orally administered anti-viral treatment and immunization system. Gut Microbes.

[174] Huang Y, Sun J, Cui X, Li X, Hu Z, Ji Q (2024). Enhancing protective immunity against bacterial infection via coating nano-Rehmannia glutinosa polysaccharide with outer membrane vesicles. J Extracell Vesicles.

[175] Bjånes E, Krishnan N, Koh T, Ngo AT, Cole J, Olson J (2025). STING-adjuvanted outer membrane vesicle nanoparticle vaccine against Pseudomonas aeruginosa. JCI Insight.

[176] Wang Z, Li M, Li W, He L, Wang L, Cai K (2025). Hybrid outer membrane vesicles with genetically engineering for treatment of implant-associated infections and relapse prevention through host immunomodulation. Adv Sci (Weinh).

[177] Du X, Dong Z, Yan Y, Gong Y, Yuan M, Ma C (2025). Immunomodulatory nanozymes eradicate intracellular infections and rescue immunoparalysis for treating multidrug-resistant bacterial sepsis. Exploration (Beijing).

[178] Qi M, Ding Q, Shi Y, Wang K, Liu J, Zhou J (2026). NIR-activated nanodisguisers for targeted bactericidal action and enhanced electron transfer in periodontitis treatment. Biomaterials.

[179] Yang X, Shi G, Lin Z, Qiu Y, Liu F, Hu K (2025). Pathogen-targeting biomineralized bacterial outer membrane vesicles for eradicating both intracellular and extracellular Staphylococcus aureus. J Control Release.

[180] Liu Q, Li B, Ma J, Lei X, Ma J, Da Y (2025). Development of a recombinant outer membrane vesicles (OMVs)-based vaccine against Helicobacter pylori infection in mice. J Extracell Vesicles.

[181] Gong Y, Hao W, Xu L, Yang Y, Dong Z, Pan P (2025). BCG-derived outer membrane vesicles induce TLR2-dependent trained immunity to protect against polymicrobial sepsis. Adv Sci (Weinh).

[182] Feng R, Xue RY, Liu C, Li GC, Deng Y, Jin Z (2025). RBD-displaying OMV nanovaccine boosts immunity against SARS-CoV-2. J Nanobiotechnology.

[183] Banerjee S, Halder P, Das S, Maiti S, Withey JH, Mitobe J (2024). Trivalent outer membrane vesicles-based combination vaccine candidate induces protective immunity against Campylobacter and invasive non-typhoidal Salmonella in adult mice. Med Microbiol Immunol.

[184] De Langhe N, Van Dorpe S, Guilbert N, Vander Cruyssen A, Roux Q, Deville S (2024). Mapping bacterial extracellular vesicle research: insights, best practices and knowledge gaps. Nat Commun.

[185] Gao Q, Zhou W, Nurxat N, Xu K, Xu Y, Li W (2025). Dynamic profiling of lipoteichoic acid (LTA) and/or lipopolysaccharide (LPS) positive extracellular vesicles in plasma as diagnostic and prognostic biomarkers for bacterial infection. Adv Sci (Weinh).

[186] Gao Q, Zhou W, Shen Z, Chen T, Hu C, Dong L (2025). Dynamic profiling of penicillin-binding protein 2a (PBP2a)-positive extracellular vesicles: implications for early diagnosis and treatment monitoring of methicillin-resistant Staphylococcus aureus infections. J Extracell Vesicles.

[187] Jung AL, Schmeck B, Wiegand M, Bedenbender K, Benedikter BJ (2021). The clinical role of host and bacterial-derived extracellular vesicles in pneumonia. Adv Drug Deliv Rev.

[188] Li Q, Ou Z, Lin J, Tang D, He B, Wu Y (2025). Specific labeling of outer membrane vesicles with antibiotic-conjugated probe reveals early bacterial infections in blood. Nat Commun.

[189] Michel LV, Gaborski T (2022). Outer membrane vesicles as molecular biomarkers for Gram-negative sepsis: taking advantage of nature's perfect packages. J Biol Chem.

[190] Gong X, Liu S, Xia B, Wan Y, Zhang S, Zhang B (2025). Oral delivery of therapeutic proteins by engineered bacterial type zero secretion system. Nat Commun.

[191] Wang Y, Hu L, Wang L, Zhang C, Shen W, Yang H (2025). Engineered Escherichia coli Nissle 1917 targeted delivery of extracellular PD-L1-mFc fragment for treating inflammatory bowel disease. Acta Pharm Sin B.

[192] Lin S, Han S, Wang X, Wang X, Shi X, He Z (2024). Oral microto-nano genome-editing system enabling targeted delivery and conditional activation of CRISPR-Cas9 for gene therapy of inflammatory bowel disease. ACS Nano.

[193] Ou Z, Situ B, Huang X, Xue Y, He X, Li Q (2023). Single-particle analysis of circulating bacterial extracellular vesicles reveals their biogenesis, changes in blood and links to intestinal barrier. J Extracell Vesicles.

[194] Li X, Qi X, Liu X, Zhu J, Hu L (2024). Lipopolysaccharide imprinted polymers for specific recognition of bacterial outer membrane vesicles. Anal Chem.

[195] Li P, Liu J, Wang Y, Li M, Gong X, Peng Z (2025). Spatiotemporal targeted delivery of biomimetic bacterial outer membrane nanoparticles for enhanced spinal cord injury repair. Adv Mater.

[196] Hamza M, Wang S, Wu H, Sun J, Du Y, Zeng C (2025). Targeting copper homeostasis: Akkermansia-derived OMVs co-deliver Atox1 siRNA and elesclomol for cancer therapy. Acta Pharm Sin B.

[197] Xiang S, Khan A, Yao Q, Wang D (2024). Recent advances in bacterial outer membrane vesicles: effects on the immune system, mechanisms and their usage for tumor treatment. J Pharm Anal.

[198] Suri K, D'Souza A, Huang D, Bhavsar A, Amiji M (2023). Bacterial extracellular vesicle applications in cancer immunotherapy. Bioact Mater.

[199] Luo Z, Cheng X, Feng B, Fan D, Liu X, Xie R (2024). Engineering versatile bacteria-derived outer membrane vesicles: an adaptable platform for advancing cancer immunotherapy. Adv Sci (Weinh).

